# Target-Specific Expression of Presynaptic NMDA Receptors in Neocortical Microcircuits

**DOI:** 10.1016/j.neuron.2012.06.017

**Published:** 2012-08-09

**Authors:** Katherine A. Buchanan, Arne V. Blackman, Alexandre W. Moreau, Dale Elgar, Rui P. Costa, Txomin Lalanne, Adam A. Tudor Jones, Julia Oyrer, P. Jesper Sjöström

**Affiliations:** 1 Department of Neuroscience, Physiology, and Pharmacology, University College London, 21 University Street, London WC1E 6DE, UK; 2 Neuroinformatics Doctoral Training Centre, Institute for Adaptive and Neural Computation, School of Informatics, University of Edinburgh, Edinburgh EH8 9AB, UK; 3 Centre for Research in Neuroscience, Department of Neurology and Neurosurgery, The Research Institute of the McGill University Health Centre, Montreal General Hospital, Montreal, QC H3G 1A4, Canada

## Abstract

Traditionally, NMDA receptors are located postsynaptically; yet, putatively presynaptic NMDA receptors (preNMDARs) have been reported. Although implicated in controlling synaptic plasticity, their function is not well understood and their expression patterns are debated. We demonstrate that, in layer 5 of developing mouse visual cortex, preNMDARs specifically control synaptic transmission at pyramidal cell inputs to other pyramidal cells and to Martinotti cells, while leaving those to basket cells unaffected. We also reveal a type of interneuron that mediates ascending inhibition. In agreement with synapse-specific expression, we find preNMDAR-mediated calcium signals in a subset of pyramidal cell terminals. A tuned network model predicts that preNMDARs specifically reroute information flow in local circuits during high-frequency firing, in particular by impacting frequency-dependent disynaptic inhibition mediated by Martinotti cells, a finding that we experimentally verify. We conclude that postsynaptic cell type determines presynaptic terminal molecular identity and that preNMDARs govern information processing in neocortical columns.

## Highlights

► NMDA receptor calcium supralinearities were imaged in cortical layer 5 neuron boutons ► Presynaptic NMDA receptor specificity is determined by postsynaptic cell identity ► Presynaptic NMDA receptors control information flow in neocortical microcircuits

## Teaser

The existence of presynaptic NMDA receptors (preNMDARs) is debated and their functional role is unclear. Buchanan et al. demonstrate that postsynaptic cell identity determines preNMDAR expression and that these receptors specifically impact short-term plasticity and information flow in neocortical microcircuits.

## Introduction

The neocortex is strikingly uniform, with extensive repetition of a limited number of circuit motifs (Douglas and Martin, 1998). But neocortical circuits are also highly diverse, consisting of a multitude of cell types with widely differing intrinsic and morphological properties (Ascoli et al., 2008; Markram et al., 2004). Specific and differential properties of synaptic connections themselves have also been reported (Galarreta and Hestrin, 1998; Markram et al., 1998; Reyes et al., 1998), for example, between neocortical pyramidal cells (PCs) or between PCs and Martinotti cell (MC) interneurons (INs). In the hippocampus, it was recently reported that postsynaptic molecular properties determine long-term plasticity in certain inhibitory cell types (Nissen et al., 2010), indicating that synaptic molecular markers may in fact define IN types (Ascoli et al., 2008).

NMDA receptors (NMDARs) are nonspecific cationic ionotropic glutamate receptors that, in the classical view, play important roles in dendritic integration (Schiller et al., 2000), excitatory transmission (Lisman et al., 2008; Salt, 1986), and coincidence detection for Hebbian plasticity (Yuste and Denk, 1995). Here, the characteristic dual dependence of NMDARs on presynaptically released glutamate and on postsynaptic depolarization is key to their proper functioning in these roles (Ascher and Nowak, 1988; MacDermott et al., 1986), which means NMDARs need to be located postsynaptically. But there is also increasing evidence for the existence of putatively presynaptic NMDARs (preNMDARs) (Corlew et al., 2008), e.g., in spinal cord (Bardoni et al., 2004), cerebellum (Casado et al., 2002; Duguid and Smart, 2004), amygdala (Humeau et al., 2003), and cortex (Berretta and Jones, 1996; Sjöström et al., 2003). These preNMDARs can impact both spontaneous and evoked neurotransmission in the short and intermediate term (Bardoni et al., 2004; Duguid and Smart, 2004; Sjöström et al., 2003) but may also play a role in the induction of long-term plasticity (Casado et al., 2002; Humeau et al., 2003; Sjöström et al., 2003). Their presynaptic location, however, is peculiar, as it seems to render, e.g., NMDAR-based detection of coincident activity in connected neurons impossible without additional signaling from the postsynaptic side (Duguid and Sjöström, 2006). This suggests that preNMDARs may also serve other, presently unknown functions, pertinent to the functioning of the microcircuit.

An important step toward understanding the functional roles of preNMDARs is to elucidate precisely where they are expressed, since a specific localization of presynaptic NMDARs to certain subsets of synapses in the microcircuit would indicate that these receptors are not there by chance, but because they are dedicated to a function in the local circuit. Directly visualizing preNMDARs, however, has proven complicated, resulting in contradictory results and disagreement (Christie and Jahr, 2009; Duguid and Sjöström, 2006). Electrophysiology experiments suggest that the expression of presynaptic NMDARs is pathway specific, with prominent expression at the L4-L2/3 path, but not at L4-L4 or L2/3-L2/3 connections (Brasier and Feldman, 2008). Indeed, internal blockade of NMDARs in recordings of monosynaptically connected L4-L2/3 pairs strongly suggest that these receptors are indeed presynaptic (Rodríguez-Moreno and Paulsen, 2008). In a recent study, however, dendritic, but not axonal, NMDAR-mediated calcium transients could be directly visualized in L5 PCs (Christie and Jahr, 2009), perhaps suggesting that, although preNMDARs are indeed located in presynaptic neurons, they are in dendrites but not axons (Christie and Jahr, 2008, 2009).

Here, we investigate the detailed localization and functional role of preNMDARs in local circuits of neocortical layer 5. We employ targeted paired recordings with mouse transgenics, two-photon laser scanning microscopy (2PLSM) of calcium signals and cell morphology, neurotransmitter uncaging, and computer simulations. We find that postsynaptic cell identity specifically determines whether functional preNMDARs are found in axonal compartments, which generate heterogeneity in synaptic terminals that may explain why these receptors have previously been difficult to detect. We also find that preNMDARs control short-term plasticity at some synapse types within L5. Finally, we propose that preNMDARs are ideally positioned to specifically control information flow in local neocortical circuits during high-frequency firing.

## Results

### NMDAR Blockade Selectively Suppresses EPSPs onto PCs but Not onto INs

Prior studies in rat neocortex indicate that blockade of preNMDARs results in a reversible reduction of excitatory neurotransmission at monosynaptic connections between L5 PCs (Sjöström et al., 2003), as well as at the L4-L2/3 path (Bender et al., 2006). L4-L4 and L2/3-L2/3 connections, however, do not respond to preNMDAR blockade (Brasier and Feldman, 2008), suggesting that preNMDAR expression may be pathway specific.

**Figure 1:**
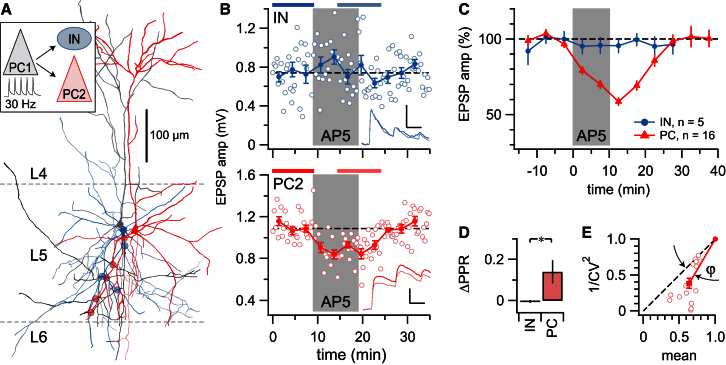
PreNMDAR Blockade Suppresses EPSPs onto PCs but Not onto INs (A) Sample triplet recording from a PC (gray; “PC1”) onto an IN (blue) as well as another PC (red; “PC2”). Circles, putative synaptic contacts; dashed lines, boundaries of neocortical layers 4, 5, and 6. For PC2 and IN morphologies, the axon is in lighter color and the dendrite is darker, but for PC1, the inverse is shown. See also Figure S2 for IN characterization. (B) PC1-IN connection is unaffected by AP5 washin during 30 Hz firing (top; 0.74mV ± 0.04mV versus 0.72mV ± 0.04mV, p = 0.722), but PC1-PC2 connection is reversibly suppressed (bottom; 1.1mV ± 0.03mV versus 0.89mV ± 0.02mV, p < 0.001). Statistics and averages before and after (inset in top and bottom panels) were taken at time periods indicated by bars at top of panels (scale bar represents 0.5mV, 20 ms). (C) Inputs onto PCs were consistently suppressed by AP5, whereas those onto INs were not (PC: after/before = 63% ± 3%, n = 15; IN: 95% ± 2%, n = 6; p < 0.001; averages taken of same periods as in B; see also Figure S1). (D) AP5-mediated suppression reduced PPR onto PCs (compare trace in bottom inset of B), consistent with a presynaptic effect. In INs, PPR was unaffected. (E) CV analysis of PC data resulted in points on or below the diagonal, consistent with AP5 acting presynaptically (angle φ = 14° ± 2°, p < 0.001) (see Experimental Procedures and Sjöström et al., 2007). For INs, CV was not affected (φ = −48° ± 40°, p = 0.25; data not shown). Error bars represent mean ± SEM.

To investigate whether preNMDARs are differentially expressed in L5, we examined in mouse visual cortex the effect of the NMDAR antagonist AP5 on monosynaptic connections from L5 PCs onto L5 INs targeted based on their distinct small rounded somata (Figure 1A). Although AP5 reliably suppressed 30 Hz excitatory postsynaptic potential (EPSP) trains at PC-PC connections (Sjöström et al., 2003), PC-IN connections were consistently unaffected (Figures 1B and 1C). The AP5-mediated suppression at PC-PC connections significantly altered the paired-pulse ratio (ΔPPR; Figure 1D), indicative of a presynaptic locus of this effect. In agreement, analysis of the coefficient of variation (CV; Figure 1E) gave rise to data points below the diagonal, which also suggests that the effect is presynaptic (cf. Sjöström et al., 2007). CV and PPR at PC-IN connections, however, were unaffected by AP5 (Figures 1D and 1E). Similar results were obtained with the GluN2B-specific antagonist Ro 25-6981 for EPSP trains onto PCs (see Figure S1 available online) (Sjöström et al., 2003).

In summary, we found that AP5 reversibly suppressed excitatory high-frequency neurotransmission, as previously shown (Bender et al., 2006; Brasier and Feldman, 2008; Sjöström et al., 2003). However, AP5 had no effect on excitatory inputs onto INs. This differential effect of AP5 was observed even when the postsynaptic PC and IN shared the same presynaptic PC (Figures 1A and 1B). Since the putative synaptic contacts of these connected pairs are interspersed along the presynaptic axon (Figure 1A), it seems unlikely that blockade of dendritic NMDARs in the presynaptic PC can explain these findings (Christie and Jahr, 2008, 2009). A more parsimonious explanation is that the NMDARs in question are located near PC-PC, but not PC-IN, synaptic terminals.

### Suppression of Neurotransmission Is Due to NMDARs in the Presynaptic PC

**Figure 2:**
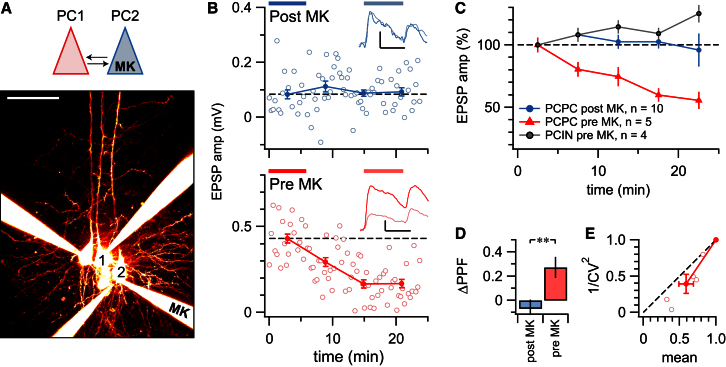
NMDAR Blockade in Pre- but Not Postsynaptic PCs Suppresses EPSPs (A) A reciprocally connected pair of PCs in which PC2 was filled with internal MK801 (“MK”) but PC1 was not. Morphology is maximum intensity projection of Alexa 594 fluorescence obtained with 2PLSM, verifying that these neurons were PCs. Scale bar represents 25 μm. (B) PC1-PC2 connection was unaffected (top; 0.08mV ± 0.02mV versus 0.09mV ± 0.01mV, p = 0.71), whereas PC2-PC1 connection was suppressed (bottom; 0.43mV ± 0.03mV versus 0.14mV ± 0.02mV, p < 0.001), indicating that pre- but not postsynaptic MK801 downregulates neurotransmission. Inset traces are averages comparing 15–21 min and 0–6 min after breakthrough. (C) Pre- but not postsynaptic MK801 consistently suppressed neurotransmission in PC-PC pairs (pre MK: 59% ± 10%, n = 5; post MK: 100% ± 4%, n = 10; p < 0.01; averaged over periods indicated in B). Presynaptic MK801 loading in PC-IN pairs was indistinguishable from post MK PC-PC pairs (120% ± 20%, n = 4; p = 0.27) but different from pre MK PC-PC pairs (p < 0.05). (D) Pre- but not postsynaptic MK801 reduced PPR in PC-PC pairs (compare inset traces in B), consistent with a presynaptic impact of presynaptic MK801. PPR in PC-IN pairs with pre MK801 was not affected (−0.09 ± 0.1, p = 0.82). (E) CV analysis of PC-PC pairs with presynaptic MK801 resulted in data points below the diagonal, confirming the presynaptic locus (φ = 10° ± 3°, p < 0.05). CV was unaffected for PC-PC pairs with postsynaptic MK801 (φ = −20° ± 24°, p = 0.42) and for PC-IN pairs with pre MK801 (φ = −60° ± 40°, p = 0.26). Error bars represent mean ± SEM.

Nonpostsynaptic NMDARs could be located close to synaptic terminals in two ways: either they are in the axon near the presynaptic terminal, or they reside in nearby compartments of a third cell type such as interneurons or glia (Dityatev and Rusakov, 2011; Duguid and Sjöström, 2006). Although the latter scenario would require transsynaptic signaling (Duguid and Sjöström, 2006), distal processes of mouse neocortical astrocytes do express NMDARs (Schipke et al., 2001). To distinguish between these two possibilities, we did paired recordings with internal MK801 in pre- or postsynaptic PCs (Figure 2A), as this drug blocks NMDARs from the inside (Bender et al., 2006; Brasier and Feldman, 2008; Rodríguez-Moreno and Paulsen, 2008).

We found that with presynaptic loading of MK801 in PC-PC pairs, 30 Hz trains of EPSPs were suppressed rapidly after breakthrough. With postsynaptic loading in PC-PC pairs or with presynaptic loading in PC-IN pairs, however, there was no such rapid downregulation of neurotransmission after breakthrough (Figures 2B and 2C). The effect of presynaptic MK801 loading in PC-PC pairs had a presynaptic locus, as assessed by the change in PPR and CV (Figures 2D and 2E).

To narrow down the IN cell type, we examined firing pattern, morphology, and synaptic properties (Ascoli et al., 2008). We found a narrow spike width, high spike threshold, and fast, nonaccommodating spiking pattern (Figure S2). PC-IN synapses were short-term depressing, and the morphology remained largely confined to L5 (Figure S2). These characteristics are consistent with the neocortical basket cell (BC) (Kozloski et al., 2001; Markram et al., 2004; Thomson et al., 2002).

These results strongly suggest that the NMDARs regulating neurotransmission are in the presynaptic cell, as previously shown for NMDARs underlying timing-dependent long-term depression (LTD) at L4-L2/3 connections (Rodríguez-Moreno and Paulsen, 2008). Our findings also lend additional support to the usage of the CV and PPR analysis methods (used below and in Sjöström et al., 2003, 2007).

### PreNMDARs Are Expressed in a Subset of Axonal Boutons

**Figure 3:**
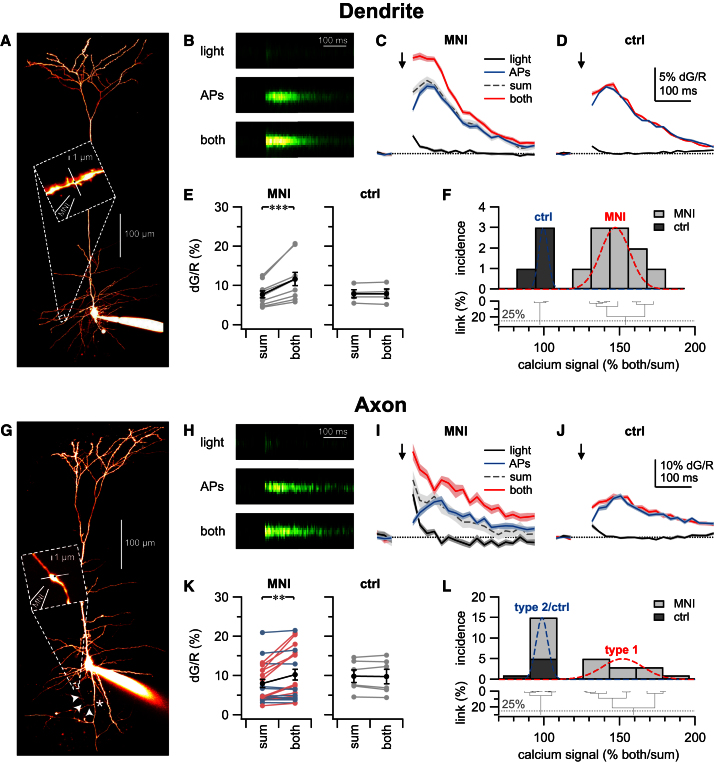
Uncaging Evokes Supralinear PreNMDAR Calcium Signals in a Subset of Boutons (A) 2PLSM maximum intensity projection of PC filled with Alexa 594, indicating the dendritic location of line scan (white continuous line) and uncaging point (black break in white line). MNI-NMDA was locally puffed right next to uncaging point (“MNI”). (B) Experimental protocol: the three conditions *light* (1-ms-long uncaging pulse), *APs* (five spikes at 30 Hz), and *both* (both simultaneously) were interleaved every 10 s and repeated six times each (see Experimental Procedures and Sjöström and Häusser, 2006). All three color maps indicate the dG change in Fluo-5F signals set to the dynamic range of the *both* condition to enable comparison. Artifact due to uncaging pulse was blanked out. (C) As expected (cf. Yuste and Denk, 1995), supralinear calcium signals (red trace) were obtained when combining uncaging (black trace) of MNI-NMDA (“MNI”) and APs (blue trace; both 12% ± 0.8% versus APs/light sum 8.9% ± 0.4%, p < 0.01). Dashed gray trace denotes arithmetic sum of APs and uncaging. Traces were smoothed in 20 ms time bins and stimulation artifact was blanked out. Arrow indicates uncaging pulse as well as start of 30 Hz burst. Traces show dG/R mean ± SEM. (D) As expected, calcium signal supralinearities were abolished when the MNI-NMDA puffing pipette was removed (“ctrl”; both 8.5% ± 0.2% versus sum 8.3% ± 0.2%, p = 0.47). For clarity, arithmetic sum trace is not shown. (E) Dendritic uncaging invariably resulted in supralinear calcium signals in the presence of MNI-NMDA or MNI-glutamate (left, “MNI”) but not in its absence (right, “ctrl”). MNI-NMDA-mediated supralinearities were blocked by internal MK801 (Figure S3). (F) As expected, hierarchical clustering independently classified control (“ctrl”) and uncaging (“MNI”) experiments into two groups based on the normalized calcium transients (both/sum). Dotted line indicates 25% linkage best cut separating the data into two classes (see Experimental Procedures). Gaussians show class mean and SD. (G) Axon collateral bifurcating off main axon (asterisk) is branching into the basal dendrites (arrowheads). Inset: axonal location of line scan, uncaging point, and MNI-NMDA puffing pipette, as in (A). (H) As with dendritic uncaging (B), conditions light, APs, and both denote interleaved averages of six line scans, with the artifact blanked out. (I) Supralinear calcium signals (red line) were recorded from the bouton in (H) when combining uncaging (black line) and APs (blue line) while puffing MNI-NMDA (“MNI”; both 20% ± 0.8% versus sum 11% ± 0.9%, p < 0.001). Binning and blanking was done as in (C). (J) The bouton calcium signal supralinearity found for the bouton in (H) vanished when the MNI-NMDA puff pipette was removed (both 12% ± 0.4% versus sum 11% ± 0.5%, p = 0.22). (K) As opposed to dendritic uncaging (E), axonal terminal calcium transients were heterogeneous, leading to supralinearities in some boutons (red), but not others (blue), in the presence of MNI-NMDA (left, “MNI”); see also Figure S4. Supralinearities were not found in the absence of MNI-NMDA (right, “ctrl”). (L) Hierarchical clustering classified the data set into two groups, and two bouton types were found: type 1 had supralinearities in response to MNI-NMDA uncaging (cf. example in G–J), whereas type 2 clustered with control experiments (see also Figure S4). Blue/red color coding for MNI in (K) is based on this clustering. Twelve of 22 boutons (55%) were classified as type 1. Error bars represent mean ± SEM.

If preNMDARs are located in synaptic terminals, it should be possible to image NMDAR-evoked bouton calcium transients with 2PLSM. NMDARs are maximally activated when simultaneously glutamate bound and depolarized (Ascher and Nowak, 1988; MacDermott et al., 1986). We therefore developed an uncaging protocol relying on this key property and tested it on dendritic NMDARs of PCs. MNI-caged NMDA or glutamate was locally puffed and uncaged with brief pulses from a violet solid-state laser, while depolarization was provided by 30 Hz trains of five action potentials (APs; see Experimental Procedures). When these two stimuli were combined, we unsurprisingly obtained supralinear calcium signals in PC dendrites (Figures 3A–3F) (Yuste and Denk, 1995). As a control, the supralinearity predictably disappeared when the caged compound was removed (Figures 3D and 3E). To show specificity, we repeatedly uncaged MNI-NMDA onto cells filled with MK801 and expectedly found that supralinearities were gradually reduced (Figure S3).

We next used the same uncaging protocol on PC boutons (Figures 3G–3L) but always with MNI-NMDA. We often found strong supralinearities (Figures 3G–3J), suggesting that there are indeed functional NMDARs in axonal compartments, close to synaptic terminals (Figures 3H and 3I). In many boutons, however, calcium signals summed linearly and were indistinguishable from controls, suggesting that not all boutons contain NMDARs (Figure 3K). In fact, we found pairs of boutons right next to each other, where one had supralinear responses to NMDA and depolarization, but the other one did not (Figure S4).

Bouton calcium signal supralinearities seemed to fall into two classes (Figure 3K), suggesting boutons with and without preNMDARs. To independently classify boutons, we employed automated clustering (see Experimental Procedures). This resulted in two classes, one of which clustered with control experiments (i.e., absence of supralinearity), in agreement with the view that preNMDAR expression is heterogeneous in L5 PC boutons (Figure 3L), but not in L5 PC dendrites (Figure 3F). In axons, the rate of finding compartments with supralinearities was also lower than in dendrites (12 out of 22 versus 10 out of 10, χ^2^ test: p < 0.05). We conclude that functional NMDARs are present in L5 PC synaptic terminals but that a substantial subset of boutons does not possess preNMDARs.

**Figure 4:**
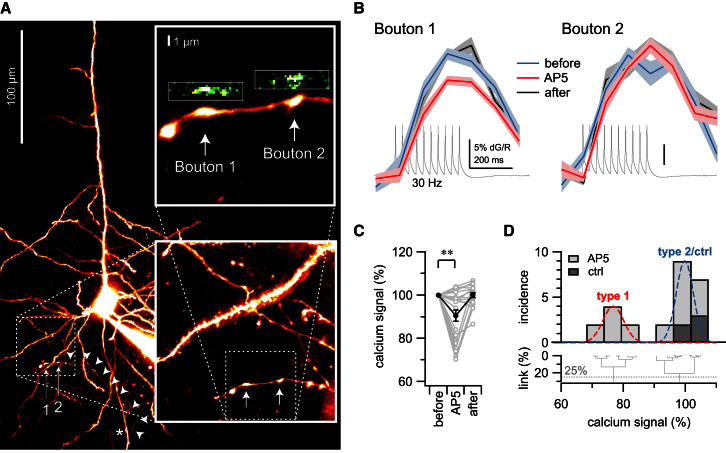
AP5 Puff Reduces Spike-Mediated Bouton Calcium Signals in a Subset of Boutons (A) An axon collateral branched off the main axon (asterisk) of a PC filled with Alexa 594 and extended up into the basal dendritic tree (arrowheads). Arrows indicate boutons 1 and 2. Bottom inset: smooth axon was readily distinguished from spiny dendrites. Dendrite running across axon was optically sectioned for clarity. Top inset: Fluo-5F revealed bouton calcium signals via 2PLSM frame scans (see Experimental Procedures). (B) When AP5 was puffed, spike-evoked calcium signals were reduced in bouton 1 (dG/R: 2.0% ± 0.1% versus 1.4% ± 0.1%, p < 0.05), but not in bouton 2 (2.5% ± 0.3% versus 2.4% ± 0.2%, p = 0.91). Image registration compensated for movements due to puff (see Experimental Procedures). (C) Although AP5 puff significantly reduced normalized calcium signals (91% ± 3%, p < 0.01 compared to 100%, n = 21), bouton responses to puffing were seemingly heterogeneous. Normalized calcium signals in mock puff controls were unaffected (102% ± 2%, p = 0.33, n = 5, data not shown). (D) Hierarchical clustering classified responses into two groups: type 1 boutons responded to AP5, whereas type 2 boutons were indistinguishable from controls. Eight of 21 boutons (38%) were classified as type 1. Error bars represent mean ± SEM.

PreNMDARs residing in L5 PC synaptic terminals might function as glutamate autoreceptors that are activated during high-frequency firing (Duguid and Sjöström, 2006; Sjöström et al., 2003). To test this hypothesis, we imaged bouton calcium signals while locally puffing the NMDAR antagonist AP5 (Figure 4). To account for tissue movements resulting from the puff, we used frame scans and automated image registration to realign imaging data (see Experimental Procedures). In keeping with uncaging experiments, NMDAR blockade resulted in significant suppression of bouton calcium transients (Figure 4C). As with the uncaging experiments (Figure S4), we occasionally found boutons right next to each other where one had NMDAR-mediated responses, but the other did not (Figures 4A and 4B). The rates of finding boutons with NMDARs using the AP5 puff and MNI-NMDA uncaging methods were indistinguishable (8 of 21 versus 12 out of 22, χ^2^ test: p = 0.28).

In summary, the existence of preNMDARs in axonal compartments most parsimoniously explains our imaging results (Figures 3 and 4), as well as the effect of loading MK801 presynaptically (Figure 2). The heterogeneity of preNMDAR expression demonstrated by our 2PLSM experiments is consistent with PC-PC, but not PC-IN, connections possessing preNMDARs (Figure 1).

### PreNMDAR Blockade Suppresses Excitation onto SOM INs

Since all the INs that we examined appeared to be BCs (Figures 1, 2, and S2), we wanted to investigate which neocortical IN types possess preNMDARs at their excitatory inputs. To better identify INs, we used transgenic mice with genetically labeled IN classes (Ascoli et al., 2008; Markram et al., 2004). Somatostatin (SOM) is one of the most specific currently available genetic markers (Toledo-Rodriguez et al., 2005), with relatively high specificity for MCs (Silberberg and Markram, 2007). We therefore targeted L5 SOM INs in slices prepared from SOM-positive transgenic mice (Oliva et al., 2000) using 2PLSM or confocal microscopy. Indeed, in targeted recordings of SOM INs, we consistently found low-threshold accommodating spiking patterns and highly facilitating excitatory inputs (Table S1), in keeping with these cells being of the MC type, as previously shown (Fino and Yuste, 2011). We therefore refer to these cells as MCs.

**Figure 5:**
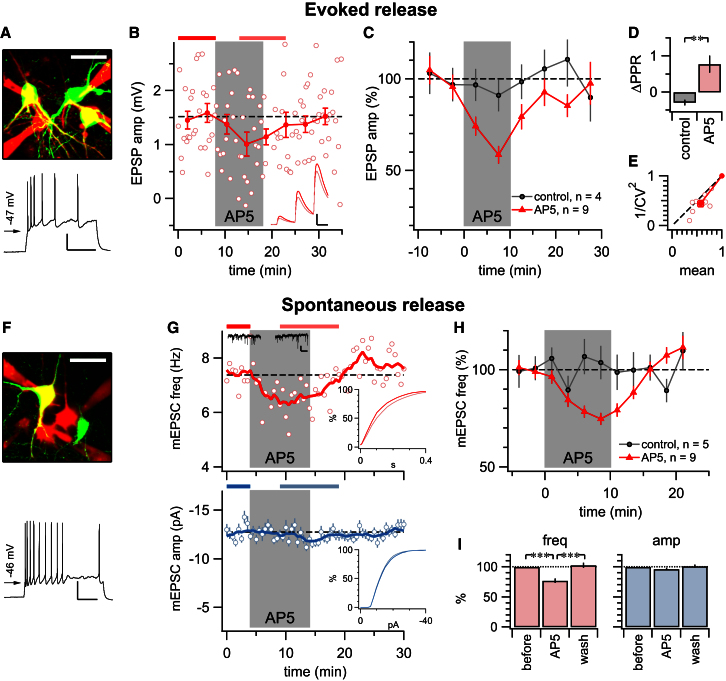
Reversible Suppression of Evoked and Spontaneous Neurotransmission onto SOM INs by PreNMDAR Blockade (A) Top: red Alexa 594 fluorescence of recorded cells overlaid on GFP fluorescence from SOM INs (Oliva et al., 2000) showing a sample mixture of targeted PCs and INs. Scale bar represents 25 μm. Bottom: firing pattern of SOM INs was consistently low threshold and accommodating. Scale bar represents 20mV, 200 ms. (B) Sample extracellular stimulation experiment in which excitatory inputs onto recorded SOM IN are reversibly suppressed by AP5 (1.5mV ± 0.1mV versus 1.1mV ± 0.1mV, p < 0.05), indicative of the existence of functional preNMDARs. Note the striking short-term facilitation, consistent with the Martinotti cell type (bottom right inset; see Silberberg and Markram, 2007). (C) Thirty hertz trains of evoked EPSPs in SOM INs were consistently suppressed by AP5 as compared to control experiments (56% ± 7%, n = 9 versus 102% ± 2%, n = 4; p < 0.001). (D) AP5 reduced PPR in SOM INs (compare inset traces in B), consistent with blockade of preNMDARs. (E) CV analysis resulted in data points on or below the diagonal, in agreement with presynaptic action of AP5 (φ = 9.3° ± 3°, p < 0.05). In controls, CV analysis did not give significant results (φ = 170° ± 100°, p = 0.2; data not shown). (F) With mEPSC recordings, GFP-positive SOM INs (Oliva et al., 2000) were targeted in parallel with PCs. Scale bar represents 25 μm. Bottom: typical SOM IN firing pattern was low threshold, accommodating, and of narrow spike width. Scale bar represents 20mV, 200 ms. (G) SOM IN recording showed reversible reduction of mEPSC frequency in AP5 (red; 6.5 ± 0.2 Hz versus 7.5 ± 0.2 Hz, p < 0.01) but no effect on amplitude (blue; −12.4 ± 0.2 pA versus −12.8 ± 0.3 pA, p = 0.32), indicating a presynaptic locus (cf. Brasier and Feldman, 2008; Sjöström et al., 2003). Top inset: sample mEPSC traces, scale bars represent 20 pA, 200 ms. Bottom inset: cumulative frequency and amplitude histograms of mEPSCs acquired during periods indicated by bars at top. Note absence of bimodality in frequency histogram, suggesting all inputs were similarly affected. (H) AP5 washin consistently reduced mEPSC frequency as compared to controls (77% ± 4%, n = 9 versus 99% ± 6%, n = 5; p < 0.01), suggesting that preNMDARs exist at most or all excitatory inputs onto SOM INs. (I) Effect of AP5 washin on mEPSC frequency was consistently reversible and never affected mEPSC amplitude significantly, similar to PCs (Brasier and Feldman, 2008; Sjöström et al., 2003). Error bars represent mean ± SEM.

Because PC-to-MC connections are highly facilitating with low probability of release (Silberberg and Markram, 2007) and since preNMDAR blockade lowers the probability of release (Figures 1 and 2; also see Sjöström et al., 2003), we hypothesized that PC-to-MC connections would not respond to NMDAR antagonism. To our surprise, however, AP5 consistently suppressed evoked neurotransmission at excitatory inputs onto MCs (Figures 5A–5C). As with PC-PC connections (Figures 1 and 2), the effect of NMDAR blockade was presynaptic by CV and PPR analyses (Figures 5D and 5E). The effects of NMDAR blockade on PC-to-MC and on PC-to-PC connections were thus similar (Figures 5A–5D versus Figures 1 and 2), even though their initial short-term dynamics are strikingly different (Markram et al., 1997; Silberberg and Markram, 2007).

Paired recordings and extracellular stimulation experiments only sample a small subset of synapses onto a given cell type. To determine whether functional preNMDARs were largely present at all excitatory inputs onto MCs, we examined spontaneous release by recording miniature excitatory postsynaptic currents (mEPSCs; see Experimental Procedures). Previous studies have shown that mEPSC frequency, but not amplitude, is lowered by AP5 even if postsynaptic NMDARs are already blocked, consistent with the existence of preNMDARs (Berretta and Jones, 1996; Brasier and Feldman, 2008; Sjöström et al., 2003). For this reason, we employed the specific reduction in mEPSC frequency without a reduction in amplitude in response to AP5 as an indicator of the presence of preNMDARs.

We first verified that AP5 reversibly reduced the frequency (to 69% ± 5% of initially 2.5 ± 0.3 Hz, p < 0.001; n = 16), but not the amplitude (to 97% ± 1% of −9.9 ± 0.3 pA, p = 0.43), of mEPSCs recorded in L5 PCs of the mouse, as previously shown in the rat (Sjöström et al., 2003). Using this approach, we found that AP5 reversibly and consistently suppressed the frequency, but not the amplitude, of spontaneous events in MCs (Figures 5F–5I). Histograms did not exhibit bimodality (cf. Figure 5G), suggesting that excitatory inputs onto MCs were homogenous with respect to preNMDAR expression. These results lend support to those obtained with evoked neurotransmission (above) and also extend our findings by suggesting that most excitatory inputs onto MCs possess preNMDARs.

### In a PV GFP Mouse Line, a Subset of INs Are Insensitive to PreNMDAR Blockade

We continued our search for the IN subtype without preNMDARs in transgenic mice. After SOM, parvalbumin (PV) is the most specific currently available genetic marker (Toledo-Rodriguez et al., 2005), chiefly labeling BC INs and chandelier cells (Ascoli et al., 2008; Markram et al., 2004; Woodruff et al., 2009). Since the INs recorded in Figures 1 and 2 had the characteristics of BCs (Figure S2), the PV marker was of particular interest. We therefore used 2PLSM to target L5 PV INs in acute slices prepared from a PV-positive GAD67-green fluorescent protein (GFP) transgenic mouse line (Chattopadhyaya et al., 2004).

**Figure 6:**
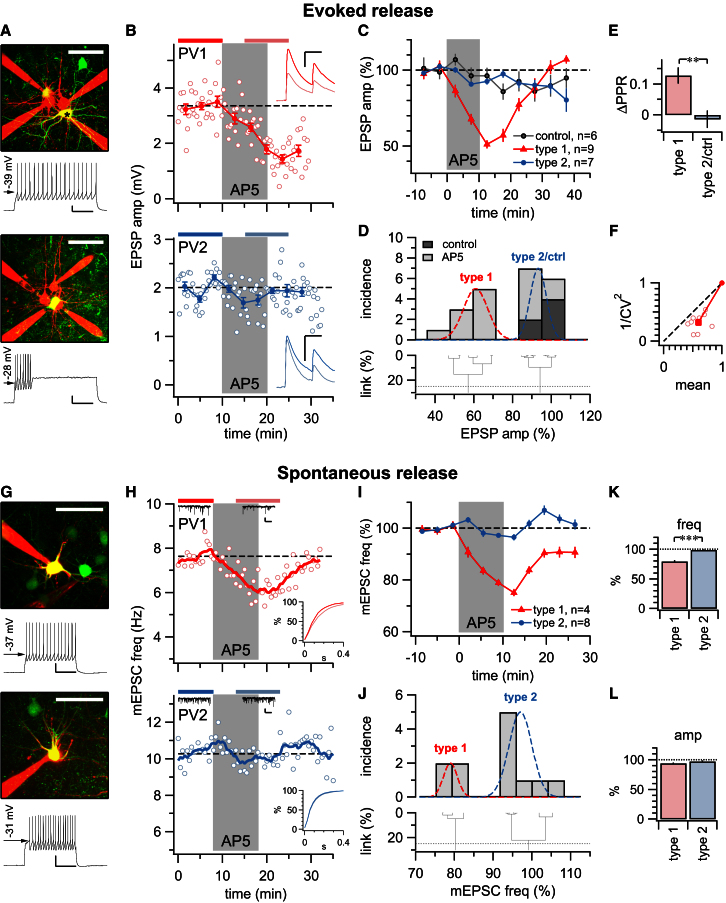
PreNMDAR Blockade Reversibly Suppresses Spontaneous and Evoked Neurotransmission onto a Subset of PV INs (A) Top: red Alexa 594 fluorescence of recorded cells overlaid on GFP fluorescence from PV INs (Chattopadhyaya et al., 2004) with recorded cell (in B, top) indicated by asterisk. Bottom: cell recorded in bottom panel of (B). Firing patterns of PV INs were consistently high threshold, nonaccommodating, and of narrow spike width. Scale bars represent 25 μm, 20mV, 200 ms. (B) Two sample paired PC-to-PV IN recordings, one of which showed EPSP suppression by AP5 (top, red; 3.4mV ± 0.1mV versus 1.8mV ± 0.1mV, p < 0.001), but the other of which did not (bottom, blue; 2.0mV ± 0.1mV versus 1.9mV ± 0.1mV, p = 0.11), suggesting that some, but not all, PC-to-PV IN connections have functional preNMDARs. Inputs were consistently short-term depressing, in agreement with the basket cell type (bottom right inset, compare to Thomson et al., 2002). (C) PV INs grouped into two types based on effect of brief AP5 washin: EPSP trains in type 1 PV INs were reversibly suppressed (61% ± 3%, n = 9, p < 0.001 compared to controls or to type 2), whereas type 2 PV INs were indistinguishable from control PV INs (91% ± 2%, n = 7 versus 95% ± 3%, n = 6, p = 0.25). (D) Hierarchical clustering (see Experimental Procedures) independently grouped PV IN AP5 responses into two classes using a 25% best cut (dotted gray line), where type 2 clustered with control experiments. Gaussians show means and SDs of the two clusters. (E) AP5 reduced PPR in type 1 PV INs compared to controls (0.13 ± 0.03, n = 9 versus −0.033 ± 0.06, n = 6, p < 0.05) and compared to type 2 PV INs (0.0030 ± 0.03, n = 7, p < 0.01), consistent with blockade of preNMDARs. As change of PPR in type 2 PV INs was indistinguishable from that of controls (p = 0.57), this data was pooled as “type 2/ctrl.” (F) Consistent with the existence of preNMDARs at inputs onto type 1 PV INs, CV analysis gave rise to data points below the diagonal, (φ = 15° ± 3°, p < 0.001). However, CV analysis did not reveal any consistent localization in type 2 PV INs (φ = −40° ± 30°, p = 0.23, data not shown) or in controls (φ = −8.0° ± 50°, p = 0.88, data not shown). (G) Top: red Alexa 594 fluorescence of GFP-positive PV INs (Chattopadhyaya et al., 2004) shown in the top panel of (H) (red). Bottom: cell recorded in bottom panel of (H) (blue). Spiking was high threshold, nonaccommodating, and of narrow spike width. Scale bars represent 25 μm, 20mV, 200 ms. (H) Top: sample type 1 PV IN (red) with reversible reduction of mEPSC frequency due to AP5 washin (7.6 ± 0.1 Hz, 6.0 ± 0.1 Hz, p < 0.001). Bottom: type 2 PV IN sample mEPSC recording for which frequency was not affected by AP5 (10.3 ± 0.13 Hz versus 10.0 ± 0.11 Hz, p = 0.15). Top inset: sample mEPSC traces, scale bars represent 20 pA, 200 ms. Bottom inset: cumulative frequency histograms. (I) The impact of AP5 washin on the frequency of spontaneous excitatory release grouped PV INs into two classes (cf. C), with type 1, but not type 2, responding. (J) As for evoked release (D), hierarchical clustering partitioned PV IN AP5 responses into two classes, indicating that type 1 has preNMDARs at most or all of its excitatory inputs, while type 2 does not have any or very few. (K and L) Although AP5 specifically reduced mEPSC frequency in type 1, but not in type 2, PV INs (80% ± 2% versus 97% ± 1%, p < 0.001), there was no such differential effect of AP5 on mEPSC amplitude (94% ± 1% versus 97% ± 2%, p = 0.146279). Error bars represent mean ± SEM.

In targeted recordings of PV INs, spiking patterns were high threshold with narrow spike width and were nonaccommodating (Table S1), consistent with BC INs (Chattopadhyaya et al., 2004; Kozloski et al., 2001; Markram et al., 2004; Thomson et al., 2002). In agreement, both excitatory inputs and inhibitory outputs of PV INs were short-term depressing (Table S1). Since the putative BC INs in Figures 1 and 2 did not appear to possess preNMDARs, we were surprised to find that in some of the PV INs, evoked EPSPs were suppressed by AP5 washin, although in other PV INs, they were not (Figures 6A–6F). Our data thus indicated the possibility that there are two types of PV INs: one with and one without preNMDARs at excitatory inputs. Because clustering separated PV IN responses to AP5 into two distinct classes (see Experimental Procedures), we denoted the corresponding PV INs as type 1 and type 2, where type 2 was indistinguishable from control experiments with mock washin (Figure 6D). As for PC-PC and PC-MC connections (Figures 1, 2, and 5), the locus of NMDAR blockade in type 1 PV INs was presynaptic according to CV and PPR analyses (Figures 6E and 6F).

The heterogeneity of preNMDAR expression at excitatory inputs onto PV INs could also be explained by the possible existence of two types of presynaptic PCs, one of which possesses NMDARs at synaptic terminals and the other of which does not. We therefore looked for preNMDARs at synapses onto PV INs by recording spontaneous neurotransmission, as this approach relatively globally samples inputs onto a recorded cell (see SOM INs above and Berretta and Jones, 1996; Brasier and Feldman, 2008; Sjöström et al., 2003). We found that the frequency of mEPSCs was reduced by AP5 in some, but not all, PV INs (Figures 6G–6L), in keeping with our results for evoked neurotransmission onto PV INs. Again, clustering segregated the data into two distinct classes (Figure 6J). Our spontaneous release experiments are most parsimoniously explained by the existence of two types of PV INs, with type 1, but not type 2, possessing preNMDARs at its excitatory inputs.

### Postsynaptic Cell Morphology Predicts PreNMDAR Expression

**Figure 7:**
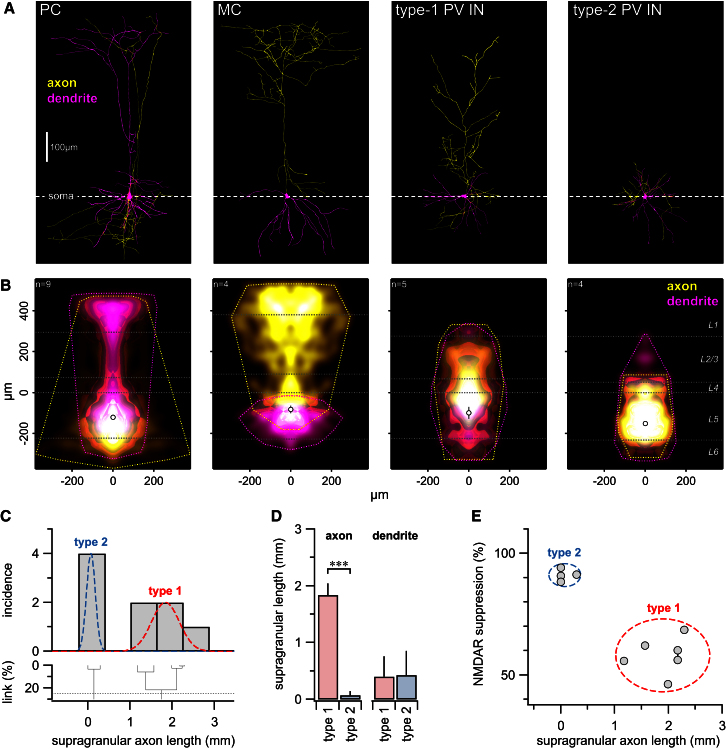
Postsynaptic Morphology Predicts PreNMDAR Expression (A) Sample morphologies of PC, MC, type 1, and type 2 PV INs, aligned on their somata (dashed line). (B) Density maps (see Experimental Procedures) show average extent of axonal (yellow) and dendritic (magenta) arborizations, while the convex hulls (yellow/magenta dotted lines) illustrate their maximum extents. Horizontal white dotted lines demarcate neocortical layer boundaries. Open circles denote somata positions (not significant [n.s.] for all comparisons). See Figures S5 and S6 for further morphometry and Table S1 for electrophysiology. (C) PV IN morphologies could be independently clustered into two types based on the amount of supragranular axon (cf. Figure S7). (D) Although axonal arbor lengths of type 1 and type 2 PV INs in the supragranular layers were different (1.8 ± 0.2 mm versus 73 ± 70 μm, p < 0.001), supragranular dendritic arborization lengths were indistinguishable (0.36 ± 0.4 mm versus 0.43 ± 0.4 mm, p = 0.96). (E) Postsynaptic axonal morphology predicted functional preNMDAR expression in those PV INs for which both electrophysiology (Figure 6, spontaneous and evoked data pooled) and morphology data were obtained. Dashed ovals represent means ± 2 SDs. Connected type 1 data points originate from one PV IN with two presynaptic PCs. Error bars represent mean ± SEM.

We next determined the morphological characteristics of the postsynaptic cell types investigated thus far: PCs, MCs, and PV INs (Figure 7A). PCs had a characteristic apical dendrite with an axon that remained largely confined to L5, although with some cells it ventured up to L1 (see Markram et al., 1997). The morphology of MCs was characteristically inverted to that of PCs, with ascending axons ramifying up to L1 and with dangling dendrites (Silberberg and Markram, 2007).

PV INs were reconstructed blind to electrophysiological type, and upon unblinding of the data set, it was clear that the axonal morphologies of the two types were distinct: type 1 PV INs had an ascending axon that reached L2/3, whereas the axonal arbor of type 2 PV INs remained in L5 (Figures 7A and 7B). In fact, PV INs could be independently clustered into two classes based on the total length of all axonal arborizations in the supragranular layers L2/3 and L1 (Figure 7C). The dendritic trees, however, did not differ (Figure 7D), suggesting that axonal, but not dendritic, branching pattern distinguishes these PV IN cell types (Ascoli et al., 2008; Markram et al., 2004).

We were concerned that the layer-specific differences in axonal arborizations between postsynaptic neuronal types in Figure 7 were the result of a 2PLSM imaging bias. However, we found that the imaged regions were indistinguishable (Figure S5). We also examined Sholl diagrams (Sholl, 1953) but found them relatively poor at distinguishing the two PV IN types, whereas the extent of supragranular axonal branching consistently separated the two PV IN types well (Figure S5).

Intriguingly, irrespective of whether the effect of AP5 or axonal supragranular layer branching was used to cluster PV INs, the same cells were grouped together (Figure 7E). In other words, the postsynaptic cell morphology consistently predicted the existence of functional preNMDARs at excitatory inputs onto individual PV INs and vice versa. Our results thus suggest that a postsynaptic cell determines the molecular composition of presynaptic terminals onto it.

Even though we used the PV IN mouse line to improve specificity compared to wild-type IN recordings (Figure 1), we unexpectedly found two PV IN types instead of one. The axonal projection pattern of type 2 PV INs—which was confined to L5—indicated that these were classical BCs. The ascending axonal arborization of the type 1 PV INs, however, has to our knowledge not been described previously in any detail (compare Kapfer et al., 2007; Kätzel et al., 2011; Thomson et al., 2002). We investigated the possibility that these were Chandelier cells (Woodruff et al., 2009) by looking for putative synaptic contacts on the axon hillocks of PCs, which were reciprocally connected with type 1 PV INs. However, putative contacts from type 1 as well as from type 2 PV INs onto PCs were perisomatically located on dendrites (Figure S6). We next asked whether type 1 and type 2 PV INs were of different ages, but this was not the case (postnatal day [P] 13.6 ± 0.9 versus 13.5 ± 1.5, p = 0.96). Indeed, type 1 and 2 PV INs were occasionally found in the same acute slice. Finally, we immunostained GFP-positive INs for PV expression. In mature animals, PV immunolabeling and GFP fluorescence unsurprisingly colocalized well (as shown before, see Chattopadhyaya et al., 2004), but at P14, a subset of GFP-positive INs did not stain for PV (Figure S7), raising the possibility that young type 1 PV INs are immature and have not yet developed PV expression.

We also compared PV INs to the wild-type INs recorded in Figures 1 and 2. We found that, morphologically (Figure S2) as well as electrophysiologically (Table S1), type 2 PV INs and wild-type INs were indistinguishable. In summary, we classify type 2 PV INs and wild-type INs as BCs and SOM INs as MCs. Cross-layer innervating type 1 PV INs, however, require further investigation to be fully classified, as their somato-dendritic target in L2/3 is presently unknown. Therefore, we do not further explore the role of this cell type here.

### PreNMDARs Reroute Information Flow in Local Circuits

**Figure 8:**
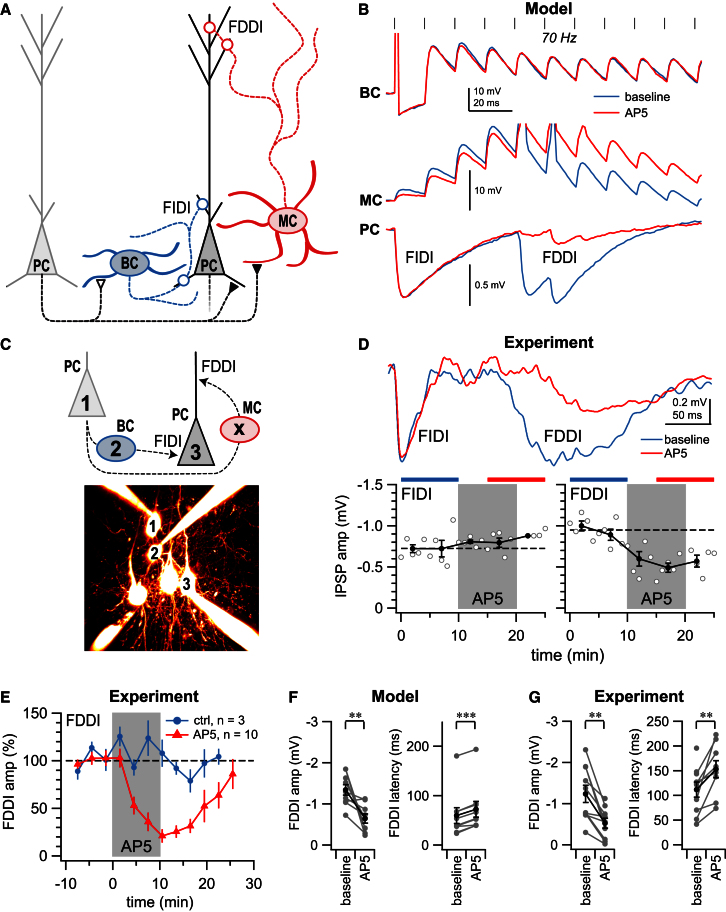
PreNMDARs Reroute Information Flow in Local Circuits during High-Frequency Firing (A) Schematic summary of our findings, showing preNMDARs at PC-PC and PC-MC connections (closed triangles), but not at PC-BC (open triangles) and BC-PC or MC-PC connections (open circles). Frequency-independent and frequency-dependent disynaptic forms of inhibition between pairs of PCs are denoted FIDI and FDDI, respectively (in keeping with Silberberg and Markram, 2007). (B) A small phenomenological network model, with tuned synaptic dynamics (Markram et al., 1998), predicted that preNMDARs impact FDDI, but not FIDI, in local circuits. As a probe for FDDI, 70 Hz presynaptic PC firing was investigated (black vertical strokes), which evoked early spiking in BCs but late spikes in MCs, resulting in a characteristic biphasic inhibitory response in postsynaptic PCs (see Silberberg and Markram, 2007). Traces show the predicted outcome before (blue) and after (red) AP5. See Tables S2 and S3 for model parameters. (C) Seventy hertz firing in PC “1” evoked both FIDI and FDDI in PC “3” when the intermediate BC “2” was subthreshold depolarized. The intermediate MC “X” was not recorded. (D) As predicted by the model (B), FDDI (right), but not FIDI (left), in PC “3” (cf. C) was affected by AP5 washin (FDDI amplitude −0.95mV ± 0.05mV versus −0.54mV ± 0.04mV, p < 0.001; FIDI amplitude −0.72mV ± 0.05mV versus −0.84mV ± 0.04mV, p = 0.12). (E) In paired PC recordings, FDDI amplitude was consistently reversibly suppressed by AP5 compared to control experiments (25% ± 9% versus 105% ± 2%, p < 0.001). (F) The computer model predicted that preNMDARs impact both FDDI amplitude (left) and latency (right) to different degrees, depending on the specifics of PC-MC short-term plasticity. Each data point corresponds to a prediction based on an individual MC recording (Figures 5A–5D; see Experimental Procedures). (G) FDDI experiments verified computer model predictions (F), showing a consistent increase in latency and suppression of amplitude due to AP5. Error bars represent mean ± SEM.

Our results indicate that PC-PC and PC-MC, but not PC-BC, connections possess preNMDARs. We also investigated the effect of AP5 on reciprocating BC-PC connections but found AP5 had no effect (data not shown), suggesting the absence of functional preNMDARs here. Based on these findings—which are summarized schematically in Figure 8A—we constructed a simple phenomenological computer model of the local circuit that incorporated measured synaptic dynamics in control and preNMDAR blockade conditions. We used this model to investigate the role of preNMDARs in the local neocortical circuit.

This simple network model predicted that preNMDARs specifically upregulate frequency-dependent disynaptic inhibition mediated by MCs (FDDI; Silberberg and Markram, 2007), while leaving BC-mediated frequency-independent disynaptic inhibition (FIDI) unaffected (Figure 8B). To test the model prediction, we repeatedly sampled the local cortical circuitry using quadruple recordings, while spiking PCs at 70 Hz (Silberberg and Markram, 2007) and while washing in AP5 to block preNMDARs. Figures 8C and 8D illustrate one such experiment for which FDDI was both reduced and delayed by AP5, while FIDI was left unaltered. Indeed, AP5 consistently and reversibly reduced FDDI amplitude and increased latency compared to control experiments (Figure 8E).

Based on the variability of synaptic dynamics measured at excitatory inputs to MCs before and after AP5 application (Figures 5A–5D), our computer model predicted that the impact of preNMDARs on FDDI would also be variable, sometimes affecting latency and/or amplitude more or less (Figure 8F). Interestingly, the impact on FDDI due to AP5 washin observed in experiments (Figure 8G) was indistinguishable from that predicted by the model (Figure 8F; p = 0.43 and 0.89 for amplitude and latency, respectively), suggesting that the contribution to FDDI from postsynaptic NMDARs at PC-MC connections is negligible, as the model had no postsynaptic NMDARs. The computer model, however, predicted that FDDI should occur earlier than what experiments revealed (onset 60 ± 16 ms, n = 9 versus 110 ± 20 ms, n = 10, p < 0.05; Figures 8F and 8G). This difference—which is due to simplifications in the model (see Experimental Procedures)—is of little or no consequence for our main finding.

To summarize, our model predicted a synapse-specific functional impact of preNMDARs on information flow in local neocortical circuits during high-frequency firing. We tested and validated this prediction experimentally. We conclude that preNMDARs are not implicated in BC-mediated FIDI but are in MC-mediated FDDI (Silberberg and Markram, 2007).

## Discussion

We find that preNMDARs are specifically expressed at a subset of synapses within a single layer of developing neocortex, which supports and elaborates on the principle that presynapse identity is governed by postsynaptic cell type (Galarreta and Hestrin, 1998; Markram et al., 1998; Reyes et al., 1998). Using 2PLSM of calcium signals in axonal boutons, we also provide direct evidence that preNMDARs are indeed in axonal compartments. Finally, by examining the impact of preNMDARs in the context of local microcircuit motifs, we discover a functional link between preNMDARs and MC-mediated FDDI (Silberberg and Markram, 2007), whereby preNMDARs upregulate FDDI during high-frequency firing. These findings are summarized in schematic form in Figure 8A. In addition, we also discover a PV IN type that mediates ascending cross-laminar inhibition to L2/3.

### The Precise Location of PreNMDARs

The existence of NMDARs in axonal compartments has been controversial. Casado and Ascher found some of the earliest electrophysiological evidence for preNMDARs at parallel fiber synapses in the cerebellum (Casado et al., 2000, 2002). Shin and Linden, however, disputed their existence because direct imaging of parallel fibers provided no evidence for NMDAR-mediated calcium signals, whereas a positive control—stellate interneuron terminal—did reveal such calcium signals (Shin and Linden, 2005; see Duguid and Sjöström, 2006). Surprisingly, however, Christie and Jahr later argued that calcium signals in stellate interneuron terminals are in fact not due to axonally but to dendritically located NMDARs that activate axonal calcium channels and thus indirectly elicit bouton calcium signals that are still NMDAR dependent (Christie and Jahr, 2008). Subsequently, Christie and Jahr also disputed the existence of NMDARs in neocortical L5 PC axons because of a lack of evidence for calcium signals in these compartments (Christie and Jahr, 2009). At neocortical L4-L2/3 synapses, meanwhile, the Paulsen team provided strong evidence for the existence of preNMDARs by loading presynaptic cells with MK801 (Rodríguez-Moreno and Paulsen, 2008) and recently used a novel caged form of MK801 to demonstrate their axonal localization (Rodríguez-Moreno et al., 2011). McGuinness et al. (2010) also reported preNMDARs in hippocampal Schaeffer collateral boutons.

Here, we combined paired recordings, pharmacology, 2PLSM imaging, and uncaging to show the existence of preNMDARs in axonal boutons of cortical neurons. We found that in L5, PC-PC and PC-BC neurotransmission are differentially affected by AP5 and that presynaptic, but not postsynaptic, MK801 dialysis impacts PC-PC neurotransmitter release, consistent with preNMDARs upregulating PC-PC neurotransmission. Taken together, the most parsimonious interpretation is that preNMDARs are located in the presynaptic cell at or near PC-PC, but not PC-BC, synapses. A corollary is that preNMDAR expression should be heterogeneous, which is exactly what we found; some, but not all, boutons showed preNMDAR-mediated supralinearities. Since the activation by NMDA defines this receptor type, and since the resulting supralinearities occurred on a millisecond timescale—precluding the possibility that supralinearities resulted from NMDARs in presynaptic dendrites (Christie and Jahr, 2008)—the most parsimonious explanation is that these signals result from preNMDARs local to the axon itself. Why Christie and Jahr (2009) did not find evidence for preNMDARs in L5 PC axons is unclear, but the heterogeneity of preNMDAR expression probably contributed.

Interestingly, our data hints at the existence of presynaptic NMDAR microdomains (Sjöström et al., 2008). It would be interesting to know whether such microdomains are located near the synaptic cleft itself or some micrometer distance away.

### Target-Specific Expression of PreNMDARs

The existence of preNMDARs is not only controversial, but also puzzling (Duguid and Sjöström, 2006). Because of their dual need for glutamate and depolarization to open (Ascher and Nowak, 1988; MacDermott et al., 1986), NMDARs have traditionally been viewed as coincidence detectors (Sjöström et al., 2008), but this requires that they be located postsynaptically. The precise function of preNMDARs therefore remains enigmatic. It has been proposed that they are essential for the induction of LTD (Casado et al., 2002; Sjöström et al., 2003) and of long-term potentiation (Humeau et al., 2003) and for the regulation of neurotransmitter release (Bardoni et al., 2004; Duguid and Smart, 2004; McGuinness et al., 2010; Sjöström et al., 2003). Our imaging experiments are consistent with preNMDARs enhancing evoked high-frequency release via calcium influx, although it remains unclear why spontaneous release is also affected: perhaps there is sufficient ambient glutamate, or perhaps preNMDARs flicker open at resting membrane potential (Sjöström et al., 2003). Regardless, preNMDARs may act as frequency filters during evoked release (Bidoret et al., 2009; Sjöström et al., 2003). A key step to elucidating the functional role of preNMDARs is to ascertain where they are specifically located, as nonrandom expression patterns imply a dedicated function.

A prior study by Brasier and Feldman (2008) suggests that preNMDARs are indeed expressed only in a subset of neocortical terminals, at the L4-L2/3 path, but not at L4-L4 or L2/3-L2/3 connections. Here, we extend these findings by showing that even intralayer preNMDAR expression is not random but specific. We also elucidate precisely which postsynaptic partners receive inputs from L5 PCs with and without preNMDARs, investigating in detail their morphology, intrinsic electrophysiological properties, and synaptic dynamics. We find that, in L5 of the visual cortex, PC connections onto other PCs as well as onto MCs have preNMDARs, but those onto BCs do not (cf. Figure 8A). Our findings thus support the view that preNMDARs are dedicated to a particular function (see below). Together with the Brasier and Feldman (2008) study, our results also suggest that synapse-specific preNMDAR expression is a general principle of developing neocortical circuits.

By recording spontaneous release and synaptically connected triplets, we tested the possibility that there are two types of L5 PCs, those with and those without preNMDARs, but this did not appear to be to the case. Our data instead favored the interpretation that postsynaptic cell type determines the molecular characteristics of presynaptic terminals. How the postsynaptic cell identity is communicated to presynaptic compartments is unclear, but this finding is in general agreement with prior studies showing that synaptic dynamics are dramatically dissimilar onto different interneuronal types, e.g., PC-MC versus PC-BC, even for connections originating from the one and same PC (Galarreta and Hestrin, 1998; Markram et al., 1998; Reyes et al., 1998) (cf. Figures 5 and 6 herein). We found, however, that the expression of preNMDARs was not directly linked to the type of short-term dynamics at any one given synapse type; PC-PC and PC-MC connections both have functional preNMDARs but very different short-term plasticity characteristics (Figures 1 and 5), while PC-BC connections depress short term much like PC-PC connections do, yet do not possess functional preNMDARs. Hence, even though preNMDARs regulate neurotransmitter release, they do not determine the type of short-term plasticity. We also found that ΔPPR did not correlate with PPR within any synapse type (data not shown), suggesting that the ability of preNMDARs to modulate presynaptic release did not depend on initial release probability.

### The Classification of Interneurons

IN classes are typically demarcated based on morphological, electrophysiological, synaptic, and genetic characteristics (Ascoli et al., 2008; Markram et al., 2004). Recent studies have in particular focused on axonal branching patterns as a means of determining IN type (e.g., Nissen et al., 2010).

Here, we discovered that nominally PV-positive INs of a transgenic mouse line (Chattopadhyaya et al., 2004) clustered into two types based on whether axons ramified in supragranular layers or not. Interestingly, these INs also clustered into the same two groups with respect to the existence of preNMDARs at excitatory inputs onto them (Figure 7E), which justifies their classification into two distinct types, even though they were otherwise similar. Because the morphological and electrophysiological classes matched up, it is unlikely that this separation into two classes was due to experimenter bias or to an artificial partitioning of an actual continuum. Although the main reason for using these transgenic mice was to improve specificity compared to wild-type animals, we thus surprisingly achieved less specificity. Perhaps this was because a subset of GFP-positive INs of this transgenic mouse (Chattopadhyaya et al., 2004) is not PV positive in young animals.

To our knowledge, the interlayer-projecting type 1 PV IN we found is a novel IN type. Although L5 MC axons also branch in supragranular layers, one important distinction compared to the type 1 PV INs is that L5 MCs chiefly impinge on apical dendrites of PCs (Silberberg and Markram, 2007). The type 1 PV IN, however, may perisomatically innervate L2/3 PCs, just like we found that they did with L5 PCs. Indeed, perhaps these type 1 PV INs provide the substrate for the recently reported neocortical ascending inhibition (Kätzel et al., 2011) (also see Kapfer et al., 2007; Thomson et al., 2002). Another distinction between type 1 PV INs and MCs is the overall shape of their axonal arborizations; type 1 PV INs did not reach L1, for example, while MCs did. The characteristics of the type 1 PV IN type thus remain to be elucidated, such as its postsynaptic partners and the postsynaptic somato-dendritic localization of its outputs. Fortunately, the type 1 PV INs constitute a substantial fraction of labeled INs in L5 of juvenile visual cortex of the PV mouse line (Chattopadhyaya et al., 2004), thus making them easy to target.

The distinction of type 1 and 2 PV INs by their axonal morphology lends additional support to this as a tool for IN classification (Ascoli et al., 2008; Markram et al., 2004; Nissen et al., 2010). The use of synaptic molecular markers such as preNMDARs for IN subtyping, however, is relatively unusual. A recent study in the hippocampus reported that the presence of long-term plasticity correlated with the type of PV IN and that this in turn was linked to the presence of postsynaptic calcium-permeable AMPA receptors (Nissen et al., 2010), which is a form of synaptic molecular marker. Synaptic molecular markers may thus help to classify INs.

### Functional Implications

Although preNMDARs are not ideally located for traditional coincidence detection, they are well situated to act as high-pass frequency filters (Bidoret et al., 2009; Sjöström et al., 2003). In this study, we focused on the selectivity of preNMDARs to high-frequency activity and examined its consequences for information flow in local circuit motifs. We found a link between specific preNMDAR expression and MC-mediated FDDI among neighboring PCs (Silberberg and Markram, 2007), whereby preNMDARs specifically help maintain FDDI in the face of high-frequency firing, while selectively leaving BC-mediated FIDI untouched. In L5 PCs, strong apical dendritic depolarization recruits local calcium channels to elicit complex high-frequency bursts that via MCs inhibit complex spike generation in neighboring PCs in vivo (Murayama et al., 2009). This is a powerful mechanism: four bursting PCs can elicit FDDI across an entire cortical column (Berger et al., 2010). We found that without functioning preNMDARs, FDDI was delayed or wiped out entirely. Nevertheless, in the intact brain, preNMDARs may have additional effects, such as on the cell-type-specific structure of cross-correlations (Silberberg et al., 2004).

The implications of our study are not restricted to short-term plasticity. We previously found that preNMDARs play a key role in LTD at L5 PC-PC synapses (Sjöström et al., 2003), which has since been supported by others (Corlew et al., 2008). It follows from the absence of preNMDARs that LTD at PC-BC connections cannot rely on the same mechanism. Perhaps synaptic plasticity learning rules vary with synapse types, which would have consequences for circuit refinement during development. Since preNMDARs themselves may be developmentally regulated (Corlew et al., 2008), such links to long-term plasticity are particularly interesting.

Because NMDARs are readily regulated—via glutamate spillover, glycine, neuromodulators, channel expression, and trafficking—the acute sensitivity of FDDI-based silencing of cortical columns to preNMDAR activation enables efficient and flexible control of activity in neocortical circuits. Yet, the role of preNMDARs in disease has been largely overlooked. For example, a central paradigm in modern schizophrenia research is based around NMDAR hypofunction. Indeed, it has been proposed that this may be due to a faulty NMDAR-based activity sensor (Lisman et al., 2008), but previous research has emphasized the postsynaptic side of the synapse. A potential link to preNMDARs thus beckons. Furthermore, dissociative drugs that block NMDARs, such as ketamine, may also act presynaptically. By virtue of its focus on the relatively overlooked preNMDARs, our study offers fresh perspective on neocortical functioning in health and disease.

## Experimental Procedures

### Electrophysiology

Procedures conformed to the UK Animals (Scientific Procedures) Act 1986 and to the standards and guidelines set in place by the Canadian Council on Animal Care, with appropriate licenses. P12–P20 mice were anesthetized with isoflurane, decapitated, and the brain was swiftly dissected in ice-cold artificial cerebrospinal fluid (ACSF: 125 mM NaCl, 2.5 mM KCl, 1 mM MgCl_2_, 1.25 mM NaH_2_PO_4_, 2 mM CaCl_2_, 26 mM NaHCO_3_, 25 mM dextrose; bubbled with 95% O_2_/5% CO_2_). Whole-cell recordings in acute visual cortex slices were carried out at 32°C–34°C (see Supplemental Experimental Procedures for details) with the following gluconate-based internal solution: 5 mM KCl, 115 mM K-gluconate, 10 mM K-HEPES, 4 mM MgATP, 0.3 mM NaGTP, 10 mM Na-phosphocreatine, and 0.1% w/v biocytin, adjusted with KOH to pH 7.2–7.4. D/L-AP5 (Sigma) was either bath applied or puffed at a concentration of 200 μM in ACSF. MK801 (Sigma) was applied at a concentration of 2 mM to standard internal solution. For 2PLSM imaging, 10–40 μM Alexa Fluor 594 and/or 180 μM Fluo-5F pentapotassium salt (Invitrogen) were added to the internal solution. INs were targeted by green GFP fluorescence detected by 2PLSM (see below) in transgenic mice specific for SOM (Jackson Laboratories, 3718; Oliva et al., 2000) or PV IN subclasses (Jackson Laboratories, 7677; Chattopadhyaya et al., 2004). Data were acquired using PCI-6229 boards (National Instruments) with custom software (Sjöström et al., 2001) running in Igor Pro 6 (WaveMetrics). Miniature EPSCs were recorded in voltage clamp at −80mV in the presence of 0.1 μM tetrodotoxin (TTX) and 20 μM bicuculline and were detected offline.

### Imaging

Workstations for 2PLSM were custom built (see Supplemental Experimental Procedures). Two-photon excitation was achieved using a MaiTai BB (Spectraphysics) or a Chameleon XR (Coherent) Ti:Sa laser, tuned to 800–820 nm for Fluo-5F and Alexa 594 or to 880–900 nm for GFP. Imaging data were acquired with PCI-6110 boards (National Instruments) using ScanImage v3.5-3.7 running in MATLAB (MathWorks) and was analyzed offline using in-house software running in Igor Pro (see below).

Uncaging was achieved using a 405 nm laser (MonoPower-405-150-MM-TEC, Alphalas GmbH). In uncaging experiments (Figures 3, S3, and S4), either 1 mM MNI-Glu or 1 mM MNI-NMDA dissolved in ACSF (see above) and supplemented with 20 mM HEPES was puffed using a patch pipette. Only MNI-NMDA was used for bouton uncaging, however.

Neurons were reconstructed from 2PLSM stacks using Neuromantic (http://www.reading.ac.uk/neuromantic) or from slices histologically processed for biocytin using Neurolucida (MicroBrightField). PV IN reconstructions were carried out blinded to electrophysiology. Quantitative analysis of morphologies was carried out using custom software written in Igor Pro, with classification using agglomerative single-linkage hierarchical clustering.

### Statistical Comparisons

Results are reported as mean ± SEM. Comparisons were made with Student’s t test for equal means, unless otherwise specified. Bonnferoni-Dunn’s method corrected for multiple comparisons. Significance levels are ^∗^p < 0.05, ^∗∗^p < 0.01, and ^∗∗∗^p < 0.001, respectively.

### Computer Modeling

The tuned model was implemented in MATLAB using adaptive exponential integrate-and-fire neurons with synapses based on a phenomenological short-term plasticity model (Markram et al., 1998). PreNMDAR blockade was simulated by fitting to AP5 data.

## Supplemental Information

Document S1. Figures S1–S7, Tables S1–S3, and Supplemental Experimental Procedures
