# Supplementary material for: Target-Specific Expression of Presynaptic NMDA Receptors in Neocortical Microcircuits
Source: Neuron. 2012 Aug 9;75(3):451–66. doi: 10.1016/j.neuron.2012.06.017 (PMC3657167; doi:10.1016/j.neuron.2012.06.017)
Supplement: Document S1. Figures S1–S7, Tables S1–S3, and Supplemental Experimental Procedures [file mmc1.pdf]

Neuron, Volume *75*

Supplemental Information

Target-Specific Expression of Presynaptic NMDA Receptors in Neocortical Microcircuits

Katherine A. Buchanan, Arne V. Blackman, Alexandre W. Moreau, Dale Elgar, Rui P. Costa, Txomin Lalanne, Adam A. Tudor Jones, Julia Oyrer, and P. Jesper Sjöström

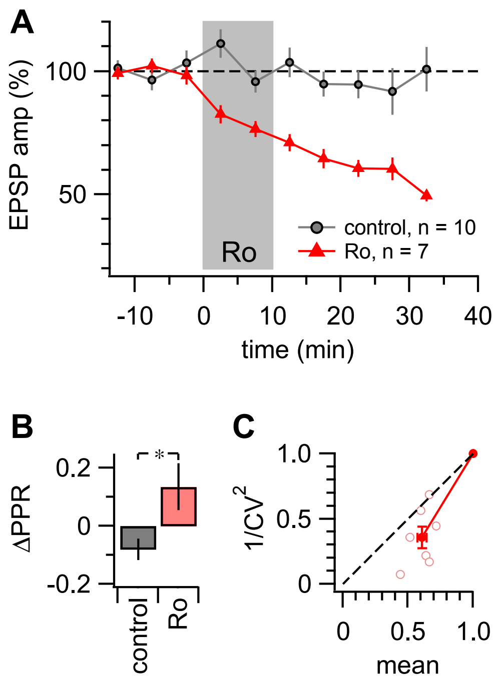

Figure S1. PreNMDARs Contain the GluN2B Subunit, Related to Figure 1

(A) The GluN2B-specific NMDAR blocker Ro 25-6981 ("Ro," Banerjee et al, 2009) consistently suppressed neurotransmission between connected L5 PC pairs compared to control experiments (61 ± 4% vs. 103 ± 5%, $p < 0.001$; Ro could not be washed out). This suggests that preNMDARs of L5 PCs have traditional coincidence detection features, as opposed to e.g. preNMDARs at the layer-4 to layer-2/3 pathway, which rely on other NMDAR subunits with non-traditional properties (Banerjee et al, 2009).

(B-C) As with AP5 and MK801, PPR and CV (Ro: $\varphi = 12 \pm 4°$, $p < 0.05$; control: $51 \pm 40°$, $p = 0.21$, not shown) were altered by Ro in agreement with a presynaptic locus.

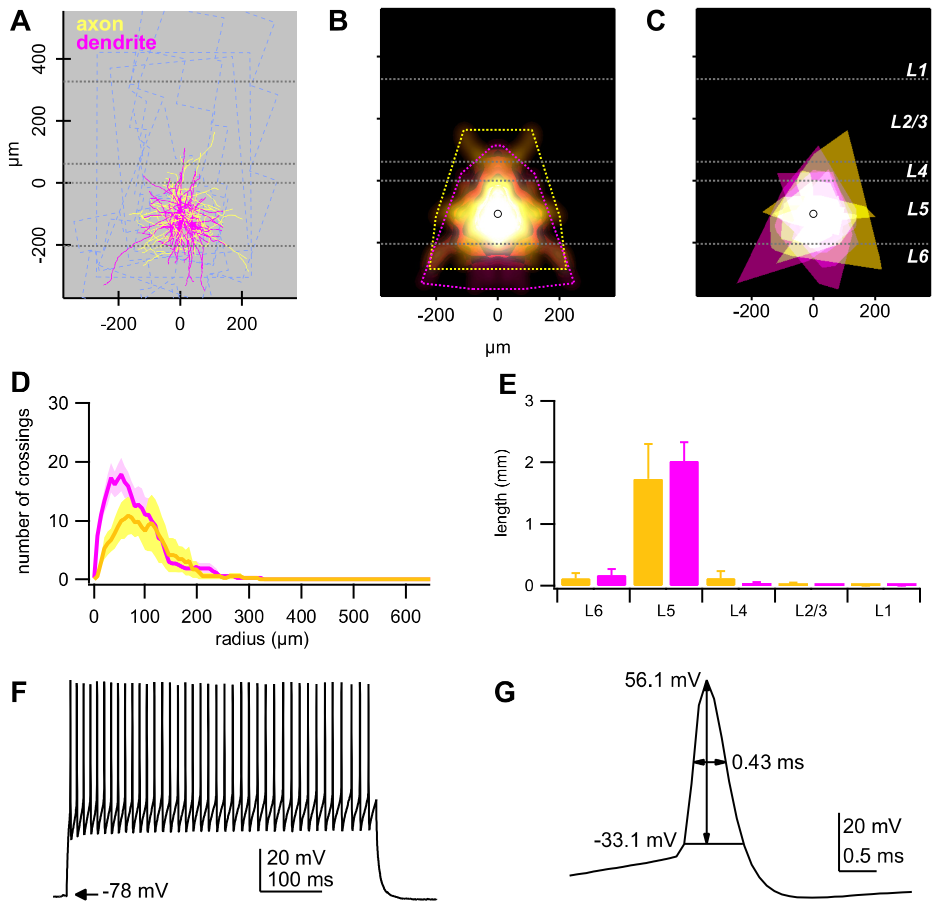

H

	IN	n	PC	n
Spike threshold (mV)	-42.04 ± 1.89	9	-45.11 ± 2.19	19
Spike height (mV)	43.42 ± 3.99	9	62.64 ± 2.63	19
Spike half-width (ms)	0.82 ± 0.21	9	1.81 ± 0.22	19
Spike afterhyperpolarization (mV)	-13.46 ± 3.16	9	6.64 ± 1.75	19
Rheobase (nA)	0.31 ± 0.11	9	0.15 ± 0.02	19
Frequency (Hz)	25.17 ± 8.57	9	9.17 ± 1.58	19
Accommodation (%)	33.2 ± 7.52	9	384.92 ± 147.14	18
CV (%)	11.97 ± 3.89	9	17.13 ± 3.22	19
V_m (mV)	-68.81 ± 3.69	9	-70.45 ± 2.17	19
R_{in} (MΩ)	153.35 ± 25.04	9	223.34 ± 26.88	19
τ_m (ms)	13.3 ± 2.9	9	29.43 ± 2.9	19
EPSP amplitude (mV)	2.37 ± 0.85	10	0.55 ± 0.11	11
EPSP PPR	-0.16 ± 0.2	10	-0.39 ± 0.11	11
IPSP amplitude (mV)	-0.85 ± 0.5	3		
IPSP PPR	-0.12 ± 0.24	3		

Figure S2. IN Characteristics Indicate a Basket Cell Type, Related to Figures 1 and 2

(A) Seven IN reconstructions are overlaid (from Figures 1 and 2), with axon in yellow and dendrite in magenta. Blue dashed lines illustrate the area imaged with 2PLSM.

(B) Density maps (see Methods) denote axonal (yellow) and dendritic (magenta) compartment densities, while the convex hulls (yellow/magenta dotted lines) illustrate

maximum extents axonal and dendritic arbours (n=7). Horizontal white dotted lines demarcate neocortical layer boundaries, while the open circle shows soma position.

(C) Overlay of axonal (yellow) and dendritic (magenta) convex hulls, illustrating the maximum arborisation extent in individual cells.

(D) The ensemble Sholl diagram shows the number of axonal (yellow) or dendritic (magenta) branches crossing a given radial distance from the soma (Sholl, 1953).

(E) Histogram of the total length of arborisation in different neocortical layers show how both axonal (yellow) and dendritic (magenta) arbours remain confined in L5.

(F) Representative rheobase spiking pattern is fast and regular, indicative of BC type.

(G) Illustration of spike threshold, half-width, and height measurements taken from the first spike in the rheobase trace in (F). Spike afterhyperpolarization was measured from the spike threshold to the post-spike minimum (not illustrated). Note the narrow spike width, characteristic of INs.

(H) Electrophysiological properties of INs in Figures 1 and 2 in comparison to a subset of PCs. Numbers are mean ± SEM. Statistically significant differences are spike height (**), spike half width (*), spike afterhyperpolarization (***), and τ_m (***).

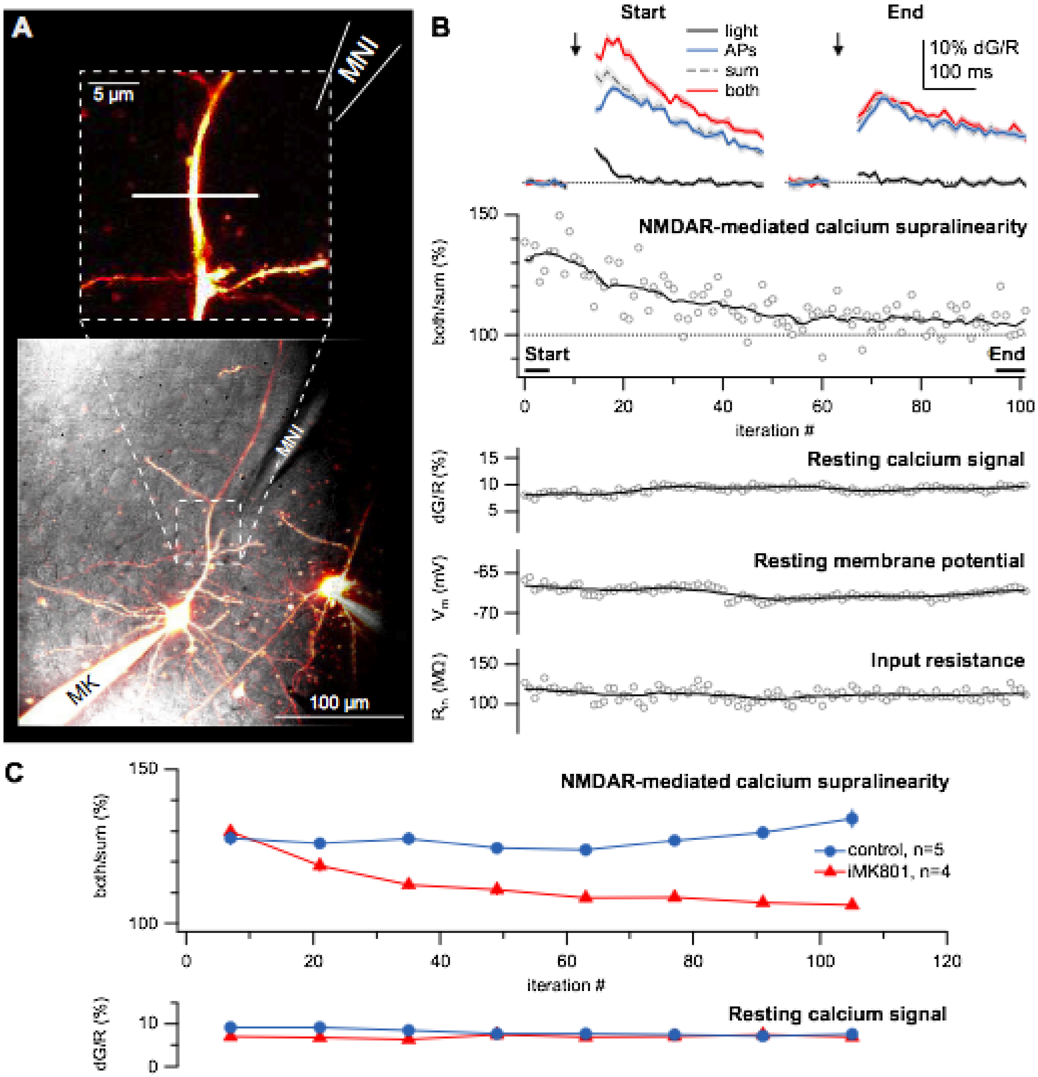

Figure S3. MNI-NMDA Specifically Acts on NMDARs, Related to Figure 3

(A) We loaded this neuron with MK801 ("MK"; Bender et al., 2006; Brasier and Feldman, 2008; Rodriguez-Moreno and Paulsen, 2008) to show the NMDAR specificity of MNI-NMDA. Ejection from puff pipette ("MNI") bends the dendrite, indicating the presence of MNI-NMDA. Line scan and uncaging carried out as for Figure 3.

(B) As expected, NMDAR-mediated dendritic calcium supralinearities (inset top left, 132% ± 2.6%, n = 6 sweeps, p < 0.001) were gradually reduced to insignificance (inset top right, 107 ± 3.3, n = 6 sweeps, p = 0.14) in the neuron in (A) dialysed with MK801, while basal calcium, membrane potential and input resistance remained stable (see Methods). Each iteration denotes the delivery of the three types of sweeps: both, light, and APs. The inter-sweep interval was two seconds.

(B) Consistent with a specific action on NMDARs by MNI-NMDA and internal MK801, dendritic calcium supralinearities remained stable in interleaved control cells (end 130% ± 7.9% vs. start 128% ± 5.6%, n = 5, p = 0.85), while supralinearities in cells loaded internally with MK801 ("iMK801") were robustly reduced (end 108% ± 1.4% vs. start 128% ± 2.9%, n = 4, p < 0.001; or p < 0.05 vs. end for controls). Internal MK801 did not affect basal calcium signals (dG/R for start 9.2% ± 1.9% vs. end 9.4% ± 1.7%, n = 5, p = 0.95).

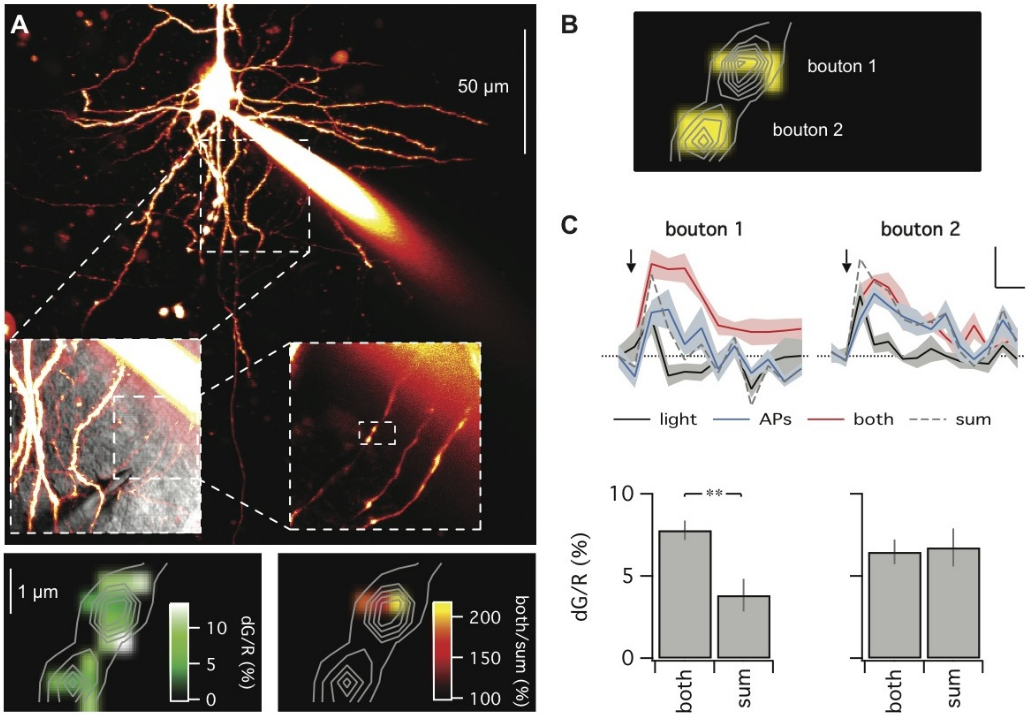

Figure S4. PreNMDAR Expression in Axonal Boutons Is Heterogeneous, Related to Figure 3

(A) Top: 2PLSM maximum intensity projection of PC filled with Alexa-594 indicating axonal position of frame scan (dotted boxes) and position of MNI-NMDA puff pipette (Dodt contrast overlay). Bottom left: Using an off-line automatic search (see Methods), significant calcium signals were detected in two boutons included in the frame scan in response to APs (5 spikes at 30Hz). Colour map indicates dG change in Fluo-5F signal. Light grey contour lines show Alexa-594 morphology. Bottom right: The same off-line search algorithm only found significant supralinear calcium responses in one of the two boutons. Colour map indicates normalized supralinearity of the *both* condition compared to the sum of APs and uncaging alone. Both AP and supralinearity colour maps were filtered to only show pixels for which $p < 0.05$.

(B) Regions of interest defining the bouton frame scans are indicated in yellow.

(C) Significant supralinear calcium signals (red line) were recorded from bouton 1 when combining uncaging (black line) and APs (blue line) while puffing MNI-NMDA. No supralinearity was observed in bouton 2, however, even though it was recorded and uncaged onto simultaneously with bouton 1.

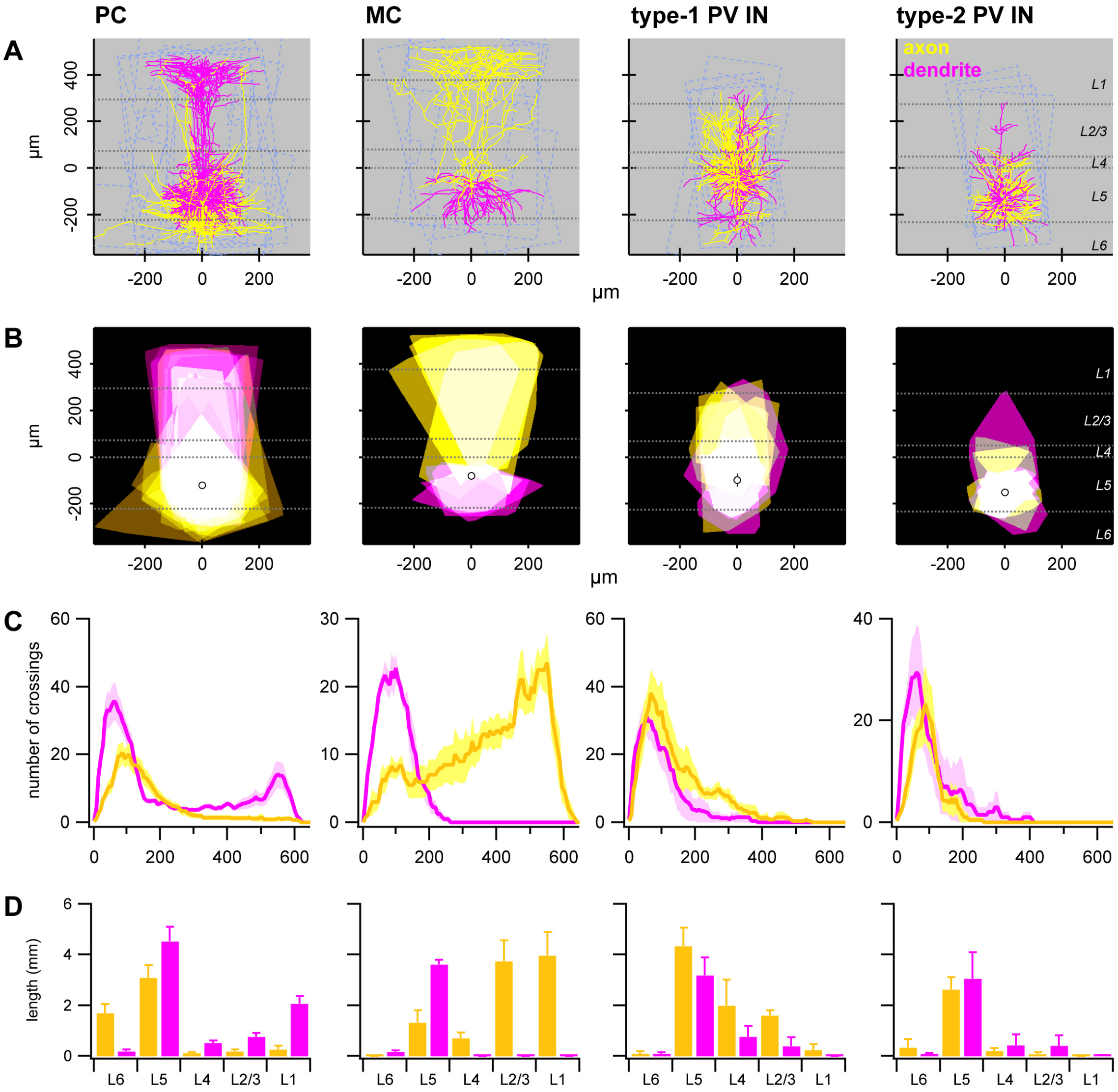

Figure S5. Morphometric Analysis, Related to Figure 7

(A) Morphologies are overlaid and aligned to show the axonal (yellow) and dendritic (magenta) arbours compared to the extent that was imaged with 2PLSM (dashed blue lines). Although axonal arborisations in upper layers were strikingly different for type-1 and type-2 PV INs (cf. Figure 6), the y-axis extent imaged was not different (720 ± 50 µm vs. 680 ± 30 µm, $p = 0.57$), indicating that this difference was not due to a 2PLSM imaging bias.

(B) Convex hulls of individual morphologies (see Methods) are overlaid to show homogeneity in maximum extent of axonal (yellow) and dendritic (magenta) arbours within cell classes. In particular, the supragranular axon hull area was different for type-1 and type-2 PV INs (55000 ± 5000 µm^2 vs. 4700 ± 3000 µm^2, $p < 0.001$), but the

supragranular dendritic hull area was not (18000 ± 10000 μm^2 vs. 11000 ± 10000 μm^2, $p = 0.67017$).

(C) Ensemble Sholl diagrams (Sholl, 1953) show the number of axonal (yellow) or dendritic (magenta) branches crossing a given radius of concentric circles centred on the soma. Although type-1 and type-2 PV INs exhibited slightly different axonal Sholl profiles, this approach was not immediately useful for distinguishing the two PV IN types, perhaps because this approach is soma- rather than layer-centric and layer-specific axonal branching is important for cell type classification.

(D) The total length of axonal (yellow) or dendritic (magenta) arborisation within a given neocortical layer was useful for classifying cells. In particular, the total length of supragranular axonal arbour found in L2/3 and L1 was used to independently classify type-1 and type-2 PV INs, as shown in Figure 7.

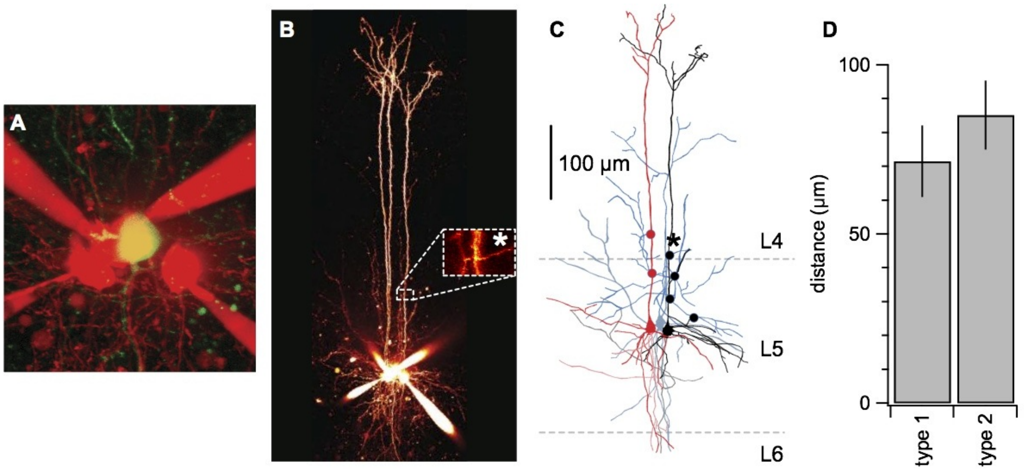

Figure S6. Perisomatic/Dendritic Location of Synapses from PV INs to PCs, related to Figure 7

(A) Flattened 2PLSM stack of three pyramidal cells and one type-1 PV IN filled with Alexa 594.

(B) The same four cells with the inset illustrating an example putative synaptic contact.

(C) Reconstruction of the three connected neurons in (A), ignoring unconnected PC for clarity. Circles: putative synaptic contacts from this type-1 PV IN onto PCs. Asterisk: contact identified in (B).

(D) The mean distance of synaptic contacts from target cell soma was indistinguishable between connections from type-1 (72 ± 11 µm; cells n = 4; connections n = 3; contacts n = 8) and type-2 PV INs (mean=85 ± 10 µm; cells n = 4; connections n = 2; contacts n = 7; p = 0.258). No synaptic contacts were found on or near the axon hillock of PCs, suggesting that these PV-positive cells were not Chandelier cells (Woodruff et al., 2009).

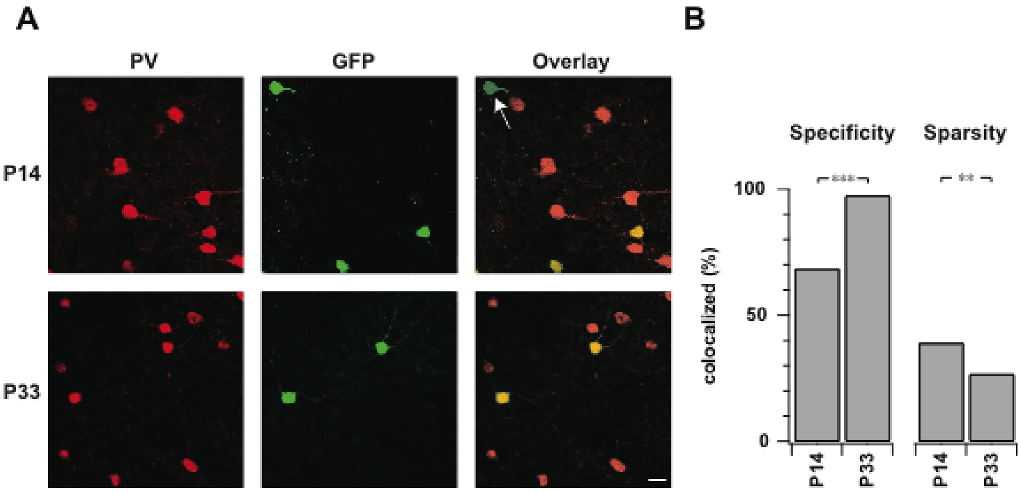

Figure S7. In Young Animals, a Subset of PV INs Are Not Positive for PV, Related to Figure 7

(A) To verify the specificity of PV-positive transgenic mice (Chattopadhyaya et al., 2004), we immuno-labelled for PV. As previously reported (Chattopadhyaya et al., 2004), labelling in mature animals was specific for PV (P33), but we surprisingly found that in immature visual cortex (P14), some “PV INs” were seemingly not PV positive (white arrow). Scale bar: 20 μm.

(B) In quantifying the staining results, it became clear that the PV-specificity of this mouse line (Chattopadhyaya et al., 2004) matured with age. In P14 animals, 70% of GFP-positive cells were PV positive, whereas virtually all were at P33 (left, “Specificity”). For simplicity, we opt to refer to immature PV-negative GFP-positive INs as PV INs, as they are genetically defined by this mouse line and may mature to PV expression later in development (Chattopadhyaya et al., 2004). With age, the percentage of GFP-positive cells out of all PV-positive cells became sparser (right, “Sparsity”). At P14, the ratio of double-labelled to all GFP-positive cells was r_{GFP} = 90/131, and for PV-positive cells r_{PV} = 90/229, with n_{animals} = 3; at P33, r_{GFP} = 87/89, r_{PV} = 87/324, with n_{animals} = 3. Significance was determined with a χ^2 test.

Table S1. Interneuron Electrophysiological Properties Indicate PV INs and Wild-type INs Are Relatively Similar, but SOM INs Are Distinct, Related to Figure 7

	Type-1 PV IN	*n*	*Type-2 PV IN*	*n*	*SOM IN*	*n*
Spike threshold (mV)	-37.96 ± 2.31	13	-36.32 ± 1.25	14	-41.81 ± 1.26	13
Spike height (mV)	40.71 ± 3.27	13	44.84 ± 2.84	14	34.45 ± 3.39	13
Spike half-width (ms)	0.67 ± 0.07	13	0.79 ± 0.07	14	1.57 ± 0.22	13
Spike afterhyp (mV)	-15.33 ± 1.63	13	-16.5 ± 1.62	14	-1.54 ± 1.67	13
Rheobase (nA)	0.4 ± 0.06	13	0.23 ± 0.04	14	0.17 ± 0.02	13
Frequency (Hz)	17.23 ± 2.08	13	16.14 ± 3.4	14	6.67 ± 1.01	13
Accommodation (%)	-21.14 ± 10.6	13	25.01 ± 9.64	14	119.73 ± 37.09	13
CV (%)	13.77 ± 5.08	13	5.82 ± 1.08	14	11.56 ± 4.24	13
V_m (mV)	-68.42 ± 2.9	13	-61.38 ± 2.86	14	-60.7 ± 1.99	13
R_{in} (MΩ)	109.59 ± 14.48	13	144.19 ± 14.48	14	171.53 ± 22.81	13
τ_m (ms)	16.33 ± 2.37	13	14.19 ± 1.55	14	24.34 ± 3.66	13
EPSP amp (mV)	1.77 ± 0.62	5	3.34 ± 1.27	5	0.19	1
EPSP PPR	-0.56 ± 0.05	5	-0.63 ± 0.03	5	1.11	1
IPSP amp (mV)	-0.41 ± 0.3	5	-0.31 ± 0.13	5	-0.09	1
IPSP PPR	-0.37 ± 0.07	5	-0.37 ± 0.13	5	-0.1	1
mEPSC amp (pA)	-17 ± 3	4	-15 ± 1	8	-12 ± 1	9
mEPSC freq (Hz)	7.3 ± 2	4	8.0 ± 0.9	8	3.4 ± 1	9

Comparison of electrophysiological properties of type-1 and type-2 PV INs indicate no major differences between the two. The only statistically significant difference is for accommodation (*). Wildtype INs (Figures 1 and 2, and Figure S2) were indistinguishable from both type-1 and type-2 PV INs. All three types — type-1 and 2 PV INs as well as wildtype INs — are thus quite similar to BCs in terms of electrophysiological properties. Note, however, that type-1 PV INs have an ascending axon (Figure 7), which is not characteristic of canonical BCs.

In contrast, the electrophysiological properties of SOM INs were saliently different. Compared to PV INs (pooled), spike threshold (*), height (***), half-width (*), afterhyperpolarization (***), V_m (**), mEPSC amplitude (*), and mEPSC frequency (**) were different. Separate comparisons to subtypes 1 and 2 of PV INs showed similar differences.

Values were extracted as described in Figure S2. Numbers are mean ± SEM. The Bonferroni-Dunn *post-hoc* correction for multiple comparisons was applied.

Table S2. Model Parameters, Related to Figure 8

**Neuron Parameters**	**PC**	**BC**	**MC**
C, membrane capacitance	132 pF	97 pF	Table S3
g_L, leak conductance	4.5 nS	6.9 nS	Table S3
E_L, resting potential	-70 mV	-70 mV	-61 mV
Δ_T, slope factor	2 mV	2 mV	2 mV
$V_{T,rest}$, threshold potential at rest	-45 mV	-37 mV	Table S3
V_{reset}, reset potential	-65 mV	-80 mV	-56 mV
$\tau_{w,ad}$, adaptation time constant	144 ms	120 ms	144 ms
a, subthreshold adaptation	4 nS	4 nS	4 nS
b, spike-triggered adaptation	80.5 pA	8 pA	Table S3
$\tau_{V,T}$, threshold potential time constant	50 ms	5 ms	50 ms
$V_{T,max}$, threshold potential after spike	18 mV	7.8 mV	-7.4 mV
**STP Parameters**	**PC1 to BC**	**BC to PC2**	**MC to PC2**
U, utilization of synaptic efficacy	0.74	0.63	0.7
F, facilitation	0.31	0.5	1
τ_F, facilitation time constant	169 ms	1000 ms	997 ms
τ_D, depression time constant	221 ms	259 ms	241 ms
A, amplitude	10 nS	-1 nS	-0.2 nS

Table S3. Model Parameters for PC-to-MC Connections, Related to Figure 8

	U		F		τ_F (ms)		τ_D (ms)		A_{SE} (nS)	C (pF)	g_L, (nS)	$V_{T,rest}$ (mV)	b (pA)
	ctrl	AP5	ctrl	AP5	ctrl	AP5	ctrl	AP5					
1	1.2e-4	1e-4	1e-4	1e-4	1000	1000	1.3	351	681	125	6	-41	155
2	3.8e-2	2e-2	5.9e-2	5.6e-2	1000	729	1000	1000	10	231	10	-40	155
3	1.8e-2	1.3e-2	6.3e-2	5.2e-2	1000	1000	1000	200	6.5	120	9	-43	155
4	0.224	0.1	0.16	0.27	998	35	399	1000	3.8	132	5	-40	100
5	0.078	0.048	0.166	0.137	932	1000	31	70	4.4	137	10	-41	155
6	0.267	0.18	0.186	0.096	361	1000	11	3.8	0.86	150	3	-45	100
7	0.306	0.105	0.38	0.422	165	880	14	34	2.43	189	9	-44	185
8	0.256	0.078	0.441	0.265	960	85	28	54	2.75	157	16	-50	155
9	0.061	0.056	0.084	0.112	1000	50.1	1000	23.4	1.45	53	2	-45	60

R^2 was 0.95 ± 0.02 and 0.94 ± 0.01 for control and AP5 experiments.

Supplemental Experimental Procedures

Slice Preparation and Basic Electrophysiology

Procedures conformed to the *UK Animals (Scientific Procedures) Act 1986* and to the standards and guidelines set in place by *the Canadian Council on Animal Care*, with appropriate licences. P12-P20 mice were anesthetized with isoflurane, decapitated, and the brain was swiftly dissected in ice-cold artificial cerebrospinal fluid (ACSF, in mM: NaCl, 125, KCl, 2.5; $MgCl_2$, 1; NaH_2PO_4, 1.25; $CaCl_2$, 2; $NaHCO_3$, 26; Dextrose, 25; bubbled with 95% O_2/5% CO_2). Near-coronal acute 300-μm-thick visual cortex slices were prepared with a MicroM or Leica VT1200S vibratome according to standard procedures (Sjöström et al., 2001). Slices were subsequently incubated at 37°C ACSF for up to one hour, after which they were allowed to cool to room temperature. All experiments were carried out at 32-34°C (Scientifica Ltd inline heater); temperature was recorded and verified off-line.

Patch pipettes (4-6 MΩ) were pulled from medium-wall capillaries using a P-97 or P-1000 electrode puller (Sutter Instruments) and were filled with internal solution (in mM): KCl, 5; K-Gluconate, 115; K-HEPES, 10; MgATP, 4; NaGTP, 0.3; Na-Phosphocreatine, 10; and 0.1% w/v Biocytin, adjusted with KOH to pH 7.2-7.4. For 2PLSM imaging (see below), 10-40 μM Alexa Fluor 594 and/or 180 μM Fluo-5F pentapotassium salt (Invitrogen, Carlsbad, CA) were added to the internal solution. To account for the higher physiological osmolality of mice compared to rats (Bourque, 2008), internal solution was adjusted from the 294 mOsm we have typically used with rat slices to 310 mOsm using sucrose, and the ACSF a corresponding amount with dextrose to 338 mOsm (cf. e.g. Sjöström et al., 2001, 2003), as we found that this increased the quality and longevity of whole-cell recordings without otherwise affecting results.

The medial side of primary visual cortex was targeted based on the presence of a granular layer 4. Whole-cell recordings were obtained in current clamp using BVC-700A (Dagan, Minneapolis, MN), MultiClamp 700B (Molecular Devices, Sunnyvale, CA), or customized ELC-03M (NPI Electronic GmbH, Tamm, Germany) amplifiers. Current clamp recordings were filtered at 5-6 kHz and acquired at 10 kHz using PCI-6229 boards (National Instruments, Austin, TX) with custom software (Sjöström et al., 2001) running in Igor Pro 6 (WaveMetrics Inc., Lake Oswego, OR) on Dell computers (Dell

Computers, Round Rock, TX). Series resistance was not compensated, but was always monitored using the custom software, as was perfusion temperature, input resistance, resting membrane potential, holding current, and EPSP amplitude, as applicable. Recordings with more than 30% change in input resistance as assessed using a 250-ms-long 25-pA hyperpolarizing current step included in every trace, or with more than 8 mV change in resting membrane potential, were discarded or truncated (Sjöström et al., 2001, 2003), but were always longer than 25 min after break-through. Junction potential was not accounted for.

Neurons were patched at 400x or 600x magnification using customized microscopes (see below; SliceScope, Scientifica Ltd; or Olympus BX51WI, Olympus, Melville, NY) with infrared video Dodt contrast (Luigs and Neumann, Ratingen, Germany; or custom-built from Thorlabs parts: a 1x telescope from LA1401 plano-convex lenses with DG20-1500-MD diffuser and a spatial filter hand cut from blackout foil, all mounted with QRC2As in 60 mm cage). L5 PCs were readily discerned by their characteristic thick apical dendrite, whereas INs were targeted by green GFP fluorescence in transgenic mice specific for SOM (Jackson Labs #3718, Oliva et al., 2000) or PV IN subclasses (Jackson Labs #7677, Chattopadhyaya et al., 2004). INs with no genetic marker (Fig. 1) were targeted based on the rounded non-pyramidal appearance of their somata as visualized with Dodt contrast. All recordings were in L5, as determined by the presence of the conspicuously large somata of L5 PCs; rare recordings for which post hoc inspection of laser-scanning Dodt contrast stacks indicated a mistaken L4 or L6 origin were discarded. Recordings denoted "wildtype" were done in the C57BL/6 strain.

Evoked Neurotransmission Experiments

As neocortical connectivity is typically sparse (e.g. 10-15% for L5-to-L5 PCs, Song et al., 2005), we employed quadruple simultaneous whole-cell recordings to increase the yield of monosynaptically connected pairs of neurons (Sjöström et al., 2001, 2003), which was further improved using software to manage multiple robotic micromanipulators (PatchStar, Scientifica). Typically, gigaohm seals were established on four target cells, after which patches were ruptured in quick succession to prevent unequal dialysis of cells, as this might otherwise have affected the outcome of experiments. Five spikes were elicited at 30 Hz using 5-ms-long current injections (0.7 - 1.4 nA) every 18 seconds in all neurons throughout the experiment. Connectivity was

assessed by averaging 10-40 such traces. To avoid accidental induction of long-term plasticity (Sjöström et al., 2001), spikes in different cells were shifted by 700 ms or more.

With extracellular stimulation experiments, stimulating electrodes (tip diameters of 2-10 µm) were filled with ACSF and were positioned in L5 under visual control, approximately 20-100 µm lateral to the somata of recorded L5 cells. Monopolar electrodes were made from standard medium-wall patch-pipette capillaries, although bipolar theta-glass electrodes were also used, with no apparent differences. Stimulation electrode position and stimulation strength were adjusted to achieve a clean stable EPSP, that is, depolarizing responses with constant short latency, a single peak, and without characteristic variable-latency down-strokes due to contamination from feedback inhibition, and that did not change in amplitude during the baseline period. At this stage, stimulation electrode strength and position was not changed for the duration of the experiment. Inhibition was thus never blocked, as we found that this was not necessary and that it would in addition adversely affect long-term quality of slices and recordings. Biphasic, 100-200-µs-long stimulation pulses were delivered in constant-voltage mode using BSI-950 (Dagan Corporation, Minneapolis, MN) or ISO-STIM 01D (NPI Electronic GmbH, Tamm, Germany) electrical stimulus isolators. Stimulation pulses replaced presynaptic spikes in paired recordings (see above). The stimulation amplitude, which was delivered and recorded by the data acquisition software, varied considerably from cell to cell, but was typically 15-40 V.

Cell identity was verified by spiking pattern and action potential width as well as morphologically by live 2PLSM of Alexa-594 red fluorescence (see below) or via post-hoc biocytin histochemistry (Vectastain ABC Elite kit, Vector Labs, Burlingame, CA) (see below, Figures 7, S2, and S5).

Evoked EPSPs were averaged during baseline and drug conditions. PreNMDAR-mediated suppression of neurotransmission was expressed in terms of the ratio of the first EPSP in a train during drug wash-in over that during the baseline. Experiments with unstable baseline, as assessed using a *t*-test of Pearson's r, were discarded. Baseline was 5-10 minutes long.

To investigate changes in short-term plasticity, we measured the paired-pulse ratio, PPR, defined as $(EPSP_2-EPSP_1)/EPSP_1$, where $EPSP_i$ is the ith EPSP in a 30-Hz train.

Although EPSP trains were always employed as a probe for preNMDARs, we found that including $EPSP_3$ and beyond in short-term plasticity analysis did not change the results (see Sjöström et al., 2007), so we only report PPR here. The change in paired-pulse facilitation, ΔPPR, was calculated as PPR_{drug} - $PPR_{baseline}$.

CV analysis was carried out as previously described (Sjöström et al., 2003, 2007). Only $EPSP_1$ in a train was used. Responses in baseline and drug conditions were separately pooled, from which mean and CV were calculated, with the CV corrected for background noise. The mean and $1/CV^2$ were normalized to the baseline period. The measure φ (see Figure 1) was defined as the angle between the diagonal and the line defined by the starting point at the coordinate (1,1) and the CV analysis endpoint (cf. Figure 1 and Sjöström et al., 2007). A presynaptic locus is thus indicated by $\varphi > 0$, while $\varphi < 0$ suggests a postsynaptic mechanism, provided that the assumptions underlying CV analysis are valid (Faber and Korn, 1991).

Spontaneous Neurotransmission Experiments

Spontaneous release was monitored by recording miniEPSCs at a holding voltage of -80 mV in the presence of 0.1 μM tetrodotoxin (TTX) and 20 μM bicuculline. These recordings were filtered at 2 kHz, and a quality criterion of <20% change in series resistance was applied in addition to input resistance change of <30%. Two sweeps of 25 seconds duration were acquired every minute, each including a 250-ms-long 5-mV hyperpolarizing step to monitor input resistance. Sweeps with rare large electrical artefacts due to e.g. electrical noise were discarded. Events were automatically detected using a customized version of the Nelson & Turrigiano software (Turrigiano et al., 1998), with detection criteria including threshold >5 pA and rise time <3 ms (cf. Sjöström et al., 2003). Events with overlap or unstable baselines were discarded. Cells with <1 Hz initial miniEPSC frequency were discarded. Experiments with unstable frequency or amplitude baseline, as assessed using a *t*-test of Pearson's r, were discarded. Baseline was 5-10 minutes long. Ensemble average time courses (e.g. Figure 5H) were created by first normalizing to the baseline period, as typically done with standard LTP experiments (see Sjöström et al., 2001).

Laser-Scanning Imaging and Uncaging Equipment

Most laser-scanning imaging was performed using three 2PLSM workstations (Denk et al., 1990), custom-built according to standard procedures (Tsai and Kleinfeld, 2009) from BX51WI (Olympus, Melville, NY) or SliceScope (Scientifica) microscopes, using hand-selected R3896 bialkali photomultipliers, C9525-51 high-voltage PSUs, and C7319 preamplifiers from Hamamatsu, or Stanford Instruments SRS570 preamplifiers. One SliceScope was fitted with an MDU (Scientifica). Scanners were Cambridge Technologies 6215H 3-mm or Thorlabs GVSM002/M 5-mm galvanometric mirrors. Photomultipliers were in epifluorescence configuration, except for in 1-photon uncaging experiments, which required a substage detector assembly configuration to allow the 405-nm laser beam access to the acute slice (see below). Two-photon excitation was achieved using a MaiTai BB (Spectraphysics) or a Chameleon XR (Coherent) Ti:Sa laser, tuned to 800-820 nm for Fluo-5F and Alexa-594, or to 880-900 nm for GFP. Gating was achieved using Thorlabs SH05/SC10 or Uniblitz LS6ZM2/VCM-D1 shutters. Laser power was manually attenuated using a polarizing beam splitter (Melles Griot PBSH-450-1300-100 or Thorlabs GL10-B with AHWP05M-980 half-wave plate) while monitoring output with a power meter (Thorlabs PM100A/S121C or Melles Griot 13PEM001/J) by picking off a fraction of the beam with a glass slide.

Some of the SOM INs were targeted with a custom-built confocal microscope, built around an Ar/Kr laser (Omnichrome 643-YOKO-A02, Melles Griot). A ZT488-491/561RPC (Chroma) polychroic reflected the 488 and 568-nm laser lines onto a 6215H scanner as well as passed the de-scanned fluorescence, which was focussed through a 150-micron pinhole (Thorlabs P150S) and collected after filtering (see below) by Hamamatsu R3896 detectors. Pinhole diameter was set to approximately the second max of the Airy function as assessed by imaging sub-diffraction-limit fluorescent beads (T14792 TetraSpeck slide, Invitrogen) onto a CCD array at the pinhole position. Confocal laser-scanning microscopy was quite adequate for targeting of genetically tagged cells for whole-cell recordings, or for assessment of morphology in Alexa-filled neurons. This imaging station was later converted to 2PLSM.

With laser-scanning Dodt-contrast imaging, the Ti:Sa or Ar/Kr laser beam was first passed through the preparation and then the Dodt tube (see above). A small component was subsequently picked off using a glass slide or 50/50 beam splitter (Thorlabs BSW17), and collected with an amplified diode (Thorlabs PDA100A-EC).

With 2PLSM, fluorescence was collected with an FF665-Di01 or -Di02 dichroic and an FF01-680/SP-25 emitter (Semrock). With both 2PLSM and confocal imaging, red/green fluorescence was selected with a t565lpxr (Chroma) or a FF560-Di01 dichroic (Semrock), a ET630/75m (Chroma) red emitter, and a ET525/50m (Chroma) or a FF01-525/45-25 (Semrock) green emitter. In uncaging experiments (see below), the 405-nm laser was blocked using a BLP01-488R-25 long-pass filter (Semrock). Visible red leakage light from the Chameleon laser was cleaned up using an LP02-664RS-25 long-pass filter (Semrock).

Imaging data was acquired with PCI-6110 boards (National Instruments) using custom-modified versions of ScanImage v3.5-3.7 (Pologruto et al., 2003) running in Matlab (MathWorks, Natick, MA), and was analysed off-line using in-house software running in Igor Pro (see below). Calcium imaging experiments commenced approximately one hour after break-through, to allow for dye equilibration. Calcium signal traces were measured as the change in green fluorescence of the calcium-sensitive dye Fluo-5F normalized to red calcium-insensitive Alexa-594 fluorescence (dG/R), either by line scans (~1 ms/line, 128-256 pixels/line, 0.5-1 s duration) or frame scans (64 pixels per line, 8 or 16 lines per frame, 8 or 16 ms/frame, 0.5-1 s duration). Electrophysiology and imaging boards were synchronized by a trig signal, which provided sub-sample precision.

Uncaging was accomplished by combining the 405-nm violet line of a 150-mW solid-state laser (MonoPower-405-150-MM-TEC, Alphalas GmbH, Göttingen, Germany) with the Ta:Sa 2PLSM beam using a Semrock FF665-Di02 dichroic, thus passing uncaging and imaging beams through the same 6215H 3-mm scanner. To backfill the objective with the 405-nm beam, a 2x telescope was built from Thorlabs parts. Parfocality of 405-nm and Ta:Sa beams, as assessed by interchangeably imaging pollen grains with either laser, was achieved by fine-adjusting this telescope. Co-alignment of 405-nm and Ta:Sa beams was verified by photolysis of CMNB-caged fluorescein (Invitrogen) or NPE-caged HPTS (Cat # 2919, Tocris) in aqueous solution (Trigo et al., 2009). Gating of the 405-nm laser was achieved using the built-in trigger, and laser power was set by adjusting the diode current, and attenuating it with a polarizing beam splitter (Thorlabs WPMH05M-405 with GL10-A). Output was monitored by picking off a fraction of the beam with a glass slide and measuring it with a power meter (Thorlabs S121C with PM100A).

Bouton Imaging Experiments

Axonal boutons were identified as axonal swellings with statistically significant calcium signals in a 50-ms-long window at the end of a 30-Hz train of action potentials compared to baseline, assessed with 5-10 sweeps. This may have selected against smaller boutons close to the diffraction limit, which may or may not contain preNMDARs.

In uncaging experiments (Figures 3, S3, and S4), either 1 mM MNI-Glu or 1 mM MNI-NMDA dissolved in ACSF (see above) was puffed using a patch pipette. Only MNI-NMDA was used for bouton uncaging, however. ACSF was supplemented with 20 mM HEPES to maintain pH. To reduce uncaging due to ambient light, MNI solutions were handled in the dark or with red LED illumination (Thorlabs LIU001), and the Olympus lighthouse was long-pass filtered at 780 nm (Thorlabs FGL780). With line scans (Figure 3), 1 or 2-ms-long uncaging pulses were used, during which the scanners were briefly fixed at the centre of the screen, corresponding to a single uncaging spot (non-scanning uncaging). With frame scanning (cf. Figure S4), the uncaging laser was on for 8 to 16 ms, during which galvanometric mirrors were continuously scanning, thus ensuring that all locations in the 8-ms frames were uncaged. Uncaging laser output power was 50-100% of max, as the built-in laser gating mechanism was faster at high power. Power was therefore further attenuated with a polarizing beam splitter to generate what corresponded to an EPSP of 1-4 mV when uncaging MNI-Glu in the proximal dendritic tree (no response was obtained, however, when uncaging in the axonal arbour). More than the 2 mW/μm^2 needed for complete uncaging of MNI with 0.1-ms-long pulse (Trigo et al., 2009) thus reached the preparation, and we furthermore used pulses that were 1 ms or longer, leading to complete photolysis of the cage. Boutons were 10-50 µm deep into the slice, which would result in laser power attenuation due to cage absorption at 405 nm, but as the cage was locally puffed this attenuation is likely considerably reduced — we assume the maximal attenuation to be 12% of initial power based on cerebellar molecular layer data (Trigo et al., 2009). Puffing is also distance dependent, but we puffed within ~10 µm, thus keeping cage concentration within 80% at the preparation (Trigo et al., 2009). We estimate uncaged NMDA and Glu peak concentration to be 0.1-1 mM, which is consistent with the observed saturating MNI-Glu EPSP responses. By uncaging the fluorescent probe NPE-HPTS (Trigo et al., 2009) while 2-photon imaging at 820 nm excitation wavelength, we estimated the uncaging cross-

section for stationary (non-scanning) uncaging spot to 6 μm diameter at half-max 10 ms after the laser flash. Uncaging laser pulse artifacts due to generation of green fluorescence were typically blanked out in imaging data; this did not affect statistical significance. We interleaved the three scan conditions *both*, *light*, and *APs* (cf. Figure 3), repeating them a minimum of six times each every 5-10 seconds (as in Sjöström and Häusser, 2006). *APs* denotes a 30-Hz train of 5 spikes alone, while *light* is the uncaging pulse alone (see above), and *both* refers to spike train and uncaging simultaneously. The dG/R traces of a scan condition were integrated of over the first 200 ms of the spike train start and averaged. Boutons with statistically significant trends in evoked calcium signals across dG/R traces (assessed by *t*-test of Pearson's r) due to e.g. physical drift or bleaching were discarded, otherwise an invalid sub or supralinearity would have been introduced.

In AP5 puff experiments (Figure 4), 10 bouton frame scans, repeated every 5-10 seconds, were acquired to establish the control calcium signal due to a train of 10 spikes at 30 Hz. In the AP5 condition, positive pressure was applied to the puffer pipette, positioned within 10 μm of the bouton, at which point 10 or more frame scans were acquired. Because of the resulting tissue movements, we employed image registration (Igor Pro) to re-centre the region of interest defining the bouton calcium signal across frame scans. Frame scans were discarded if there was excessive z-axis movement or if image registration failed. In the washout condition, pipette pressure was released, the pipette removed, and frame scans were repeated. Bouton calcium signal comparisons were based on integrating dG/R over 667 ms from the start of the spike train. To account for bleaching, frame scans were discarded if baseline green fluorescence increased significantly with time, determined by a *t*-test of Pearson's r. Frame scans were also discarded if electrophysiology did not satisfy quality criteria (change in R_{in} < 30% and in V_m < 8 mV, as above), or if the L5 PC was intrinsically bursting.

For increased visibility, calcium traces in Figures 3, 4, S3, and S4 are filtered versions of sweep averages. However, statistics were carried out on individual raw data sweeps.

Pharmacology

D/L-AP5 (Sigma) was either bath applied or puffed at a concentration of 200 μM in ACSF. Ro 25-6981 maleate (Tocris) was washed in at 0.5 μM concentration. MK801 (Sigma) was applied at a concentration of 2 mM to standard internal solution. To avoid

accidental blockade of NMDARs while approaching cells for patching, positive pressure was minimized. Furthermore, to reduce the amount of time that MK801-containing pipettes were in the slice, only one of four cells in a quadruple recording was patched with the blocker, and that cell was always patched first.

Cell Morphological Analysis

Images of cells (e.g. Figure 2) are pseudo-coloured maximum intensity projections of red Alexa-594 stacks acquired with 2PLSM, 3D-median filtered in MacBiophotonics ImageJ (www.macbiophotonics.ca). Neurons were reconstructed from 2PLSM stacks using Neuromantic (http://www.reading.ac.uk/neuromantic/), or from slices histologically processed for biocytin (see above) using Neurolucida (MicroBrightField, Magdeburg, Germany) with a 100x objective. PV IN reconstructions were carried out blinded to electrophysiology results. With Neuromantic, the boundaries of neocortical layers were identified from the simultaneously acquired laser-scanning Dodt contrast channel, by the presence of conspicuous L5 PCs with large somata and thick apical dendrites, a characteristically slightly darker granular layer 4, and a layer 1 largely devoid of cells. Putative synaptic contacts in e.g. Figure 1 were identified using 2PLSM imaging of Alexa-filled cells by axonal-dendritic crossover points with submicron separation (as in Sjöström and Häusser, 2006).

Quantitative study of digital reconstructions—such as calculation of Sholl diagrams (Sholl, 1953), convex hulls, density maps, etc—was carried out using custom software written in Igor Pro. To create density maps, morphologies were rotated a small amount (mean -4 ± 2°) around the soma to align the location of the pial surface in the "straight up" position, centred on the L4/L5 boundary, and the density map was then calculated. Each compartment of a digital reconstruction was approximated by a two-dimensional Gaussian aligned on the compartment XY centre, with amplitude proportional to compartment length, and sigma set to a constant 25 μm. These Gaussians were then summed up, thus making density maps a form of smoothed morphology cross-section 2D projection. The density map of an individual reconstruction was normalized, to permit averaging across reconstructions. Axons and dendrites for a given type were density mapped separately and these ensemble maps were normalized, gamma corrected (γ=1/2) to better visualize weak densities, assigned a colour lookup table, and merged by logical OR. Colour maps in e.g. Figure 7B are thus in arbitrary units but

comparable across cell and compartment type. Symmetry in density maps arises from mirroring of reconstructions, but all statistical comparisons were carried out on non-mirrored data.

Convex hulls of individual reconstructions were separately constructed from 2D-projections of axonal and dendritic arbours, using a Jarvis walk. Ensemble convex hulls in Figure 7B, however, are convex hulls of all convex hulls of a given type, including their mirror images. Sholl analysis (cf. Sholl, 1953) was done by re-aligning reconstructions on somata, converting to radial coordinates, and—moving in 6.5-µm steps from $r = 0$ and up, while counting the number of compartments straddling a given radius r. Sholl diagrams were then averaged without normalization.

Data Clustering

Data was automatically classified using in-house agglomerative single-linkage hierarchical clustering software running in Igor Pro, implemented according to standard procedures (Everitt et al., 2011). The squared Euclidian distance was employed as linkage metric. A 25% linkage threshold, as normalized to the greatest separation in the data set, was used as a best-cut selection criterion for the number of found clusters (compare Everitt et al., 2011; Kozloski et al., 2001). The linkage percentage units do thus not directly correspond to percentage units on the x axis (e.g. in Figure 3), because dendrograms were normalized to the greatest separation. The fuzzy c-means clustering procedure provided in Igor Pro 6.2 was also used and always gave the same classification of data points, although this implementation finds two clusters by default.

Antibody Labelling

Anesthetized mice were perfused transcardially with 4% paraformaldehyde in phosphate buffer, pH 7.4. A vibratome (DSK DTK-1000) was used to cut 60-µm thick coronal sections from visual cortex. Sections were blocked in 10% NGS and 1% Triton, then incubated overnight at 4°C with primary antibodies: Alexa 488- conjugated GFP (1:2000, Invitrogen), PV (monoclonal antibody; 1:1000; Sigma, St. Louis, MO) in 10% NGS and 0.1% Triton. Subsequently, sections were incubated with Alexa 568 conjugated goat IgG (1: 800; Invitrogen) and mounted.

Images from layer 5 of V1 were acquired using 20X air or 40X oil immersion objectives with a confocal microscope (Leica SP1). No bleed through between 488 and 568 channels was observed. Scans were collected in sequential mode and later merged. The same settings were used for acquisition of all samples. Images were saved as TIF files and analyzed with MacBioPhotonics ImageJ.

Statistical Comparisons

Results are reported as mean ± SEM. Comparisons were made using unpaired Student's *t*-test for equal means, unless otherwise specified. If equality of variances F test gave $p < 0.05$, the unequal variances *t*-test was used. Bonnferoni-Dunn's method was used to correct post hoc for multiple comparisons. Wilcoxon-Mann-Whitney's non-parametric test typically gave similar significance levels. Statistical test were carried out in Igor Pro or in Microsoft Excel. Significance levels $p < 0.05$, $p < 0.01$, and $p < 0.001$ are denoted by one, two, and three asterisks, respectively, while n.s. denotes lack of significance. To avoid the possibility of type 1 and 2 statistical errors, at least three animals were used for each group studied, and typically $n_{cell} = n_{animal}$.

Computer Modelling

The computer network model (Figure 8) was implemented in Matlab using adaptive exponential integrate-and-fire neurons (Brette and Gerstner, 2005). All parameters were either obtained from published literature or tuned to average properties from our experimental data (see below and Table S2). For MCs however, synaptic and intrinsic properties were extracted from individual recordings, with adaptation qualitatively matched to data (see below and Table S3). Synapses were modelled using the Markram & Tsodyks short-term plasticity model (Tsodyks and Markram, 1997) extended with facilitation (Markram et al., 1998). In our implementation, we consider the amount of facilitation as being an extra parameter, F, while Markram et al. 1998 assumed this to be equal to the baseline release probability. With our modification, the model provides a better fit to facilitating synapses. EPSP amplitudes were extracted from experiments by subtracting fitted exponentials to account for temporal summation (see Sjöström et al., 2007). The short-term plasticity parameters U, F, τ_D, and τ_F were numerically fitted to a subset of recordings through minimization of the mean-squared error between the normalized EPSPs amplitudes obtained from the averaged traces and those obtained from the model output, by combining a genetic algorithm with constrained gradient

descent to find the global MSE minimum. Optimization was repeated 10 times to ensure convergence. For PC-MC connections, preNMDAR blockade was simulated using the model fitted to the data recorded after the drug wash-in. In the network model, all other connections — PC-BC, BC-PC, and MC-PC — were unaffected in simulated preNMDAR block. BC-PC and MC-PC connections were tuned to a single representative synapse of said type, and thus had short-term depression. Synaptic strengths at connections originating from model PCs were tuned to trigger one or a few action potentials in postsynaptic cells due to a 15-spike 70-Hz train of presynaptic spikes (see Figure 2 in Silberberg and Markram, 2007). This usage of relatively large connective strengths thus approximates a scenario where small groups of cells are involved (Kapfer et al., 2007), with each group being represented by a single cell in our model. To obtain a degree of realistic variability in simulations, Gaussian noise with σ = 50 pA was added to postsynaptic responses and results were averaged over 50 trials.

SUPPLEMENTAL REFERENCES

Banerjee, A., Meredith, R.M., Rodriguez-Moreno, A., Mierau, S.B., Auberson, Y.P., and Paulsen, O. (2009). Double Dissociation of Spike Timing-Dependent Potentiation and Depression by Subunit-Preferring NMDA Receptor Antagonists in Mouse Barrel Cortex. Cereb Cortex.

Bourque, C.W. (2008). Central mechanisms of osmosensation and systemic osmoregulation. Nat Rev Neurosci *9*, 519-531.

Brette, R., and Gerstner, W. (2005). Adaptive exponential integrate-and-fire model as an effective description of neuronal activity. Journal of Neurophysiology *94*, 3637-3642.

Denk, W., Strickler, J.H., and Webb, W.W. (1990). Two-photon laser scanning fluorescence microscopy. Science *248*, 73-76.

Everitt, B.S., Landau, S., Leese, M., and Stahl, D. (2011). Cluster Analysis, 5 edn (Chichester, UK: John Wiley & Sons Ltd).

Faber, D.S., and Korn, H. (1991). Applicability of the coefficient of variation method for analyzing synaptic plasticity. Biophysical journal *60*, 1288-1294.

Kozloski, J., Hamzei-Sichani, F., and Yuste, R. (2001). Stereotyped position of local synaptic targets in neocortex. Science *293*, 868-872.

Markram, H., Wang, Y., and Tsodyks, M. (1998). Differential signaling via the same axon of neocortical pyramidal neurons. PNAS *95*, 5323-5328.

Pologruto, T.A., Sabatini, B.L., and Svoboda, K. (2003). ScanImage: flexible software for operating laser scanning microscopes. Biomed Eng Online *2*, 13.

Sholl, D.A. (1953). Dendritic organization in the neurons of the visual and motor cortices of the cat. J Anat *87*, 387-406.

Silberberg, G., and Markram, H. (2007). Disynaptic inhibition between neocortical pyramidal cells mediated by Martinotti cells. Neuron *53*, 735-746.

Song, S., Sjöström, P.J., Reigl, M., Nelson, S., and Chklovskii, D.B. (2005). Highly nonrandom features of synaptic connectivity in local cortical circuits. PLoS biology *3*, e68.

Trigo, F.F., Corrie, J.E., and Ogden, D. (2009). Laser photolysis of caged compounds at 405 nm: photochemical advantages, localisation, phototoxicity and methods for calibration. J Neurosci Methods *180*, 9-21.

Tsai, P.S., and Kleinfeld, D. (2009). In vivo two-photon laser scanning microscopy with concurrent plasma-mediated ablation: Principles and hardware realization. In Methods for In Vivo Optical Imaging, R. Frostig, ed. (CRC Press), pp. 59-115.

Tsodyks, M.V., and Markram, H. (1997). The neural code between neocortical pyramidal neurons depends on neurotransmitter release probability. PNAS *94*, 719-723.

Turrigiano, G.G., Leslie, K.R., Desai, N.S., Rutherford, L.C., and Nelson, S.B. (1998). Activity-dependent scaling of quantal amplitude in neocortical neurons. Nature *391*, 892-896.
